# Supplementary material for: Chewing, dentition and tooth wear in Hippopotamidae (Hippopotamus amphibius and Choeropsis liberiensis)
Source: PLoS One. 2023 Oct 4;18(10):e0291825. doi: 10.1371/journal.pone.0291825 (PMC10550173; doi:10.1371/journal.pone.0291825)
Supplement: S1 File — (PDF) [file pone.0291825.s001.pdf]

PLoS One – Supplementary Material

Chewing, dentition and tooth wear in Hippopotamidae (*Hippopotamus amphibius* and *Choeropsis liberiensis*)

Annika Avedik, Marcus Clauss*

Clinic for Zoo Animals, Exotic Pets and Wildlife, Vetsuisse Faculty, University of Zurich, Winterthurerstr. 260, 8057 Zurich, Switzerland

Supplementary text: age dependence, cranium and mandible width, eruption sequences

Supplementary text: deciduous teeth

Table S1

Figure S1-S38

Supplementary references

Supplementary text: age dependence, cranium and mandible width, eruption sequences
With age, the length of the skull and the mandible increased **(Fig. S23-S24).** Both measures were tightly related to each other, but in the relationship with age, there was a dichotomy in common hippos **(Fig. S23-S24).** This dimorphism was also evident in the length of the incisors (**Fig. S25**) and the canines (**Fig. S26**). While it is tempting to ascribe this to sexual dimorphism, the few specimens of known sex do not unambiguously support this notion, in line with the general observation that sexual dimorphism, though present, is not very pronounced in common hippos [1]. Rather, the results support the notion that growth in hippos is very variable, as was reported for their canines by Matthes [2].

When comparing maxilla or mandible width to the width of anterior teeth or the space between the anterior teeth, most scaling relationships for the cranium included linearity within common hippos and across the two species, whereas for the mandible, the sum of anterior tooth width increased more-than-linearly with mandible width in common hippos whereas the space between the anterior teeth nearly stayed constant across body sizes; across both species, the tooth width scaling did not exclude linearity, but that of the interdental spaces was below linearity **(Fig. S27)**. This indicates that in common hippos, the lower first incisors and/or canines increase with positive allometry with mandible width.

Plotting tooth height against the age classes reflects tooth wear **(Fig. S28-29)**.

The dental anatomy of the deciduous teeth and the eruption pattern of the permanent teeth in the common hippo have been published for the lower jaw [3, 4]. More recently, the full eruption sequence for common and pygmy hippos have been published in the supplementary material of Gomes Rodrigues et al. [5].

Hippopotamus amphibius
Upper jaw: $dc\text{-}dp^2\text{-}dp^3$ (→) dp^1 → dp^4 → C,M^1 → P^2 → P^3,M^2 → M^3 (→) P^4 [5]
Lower jaw: dc → dp_1,dp_2,dp_3 (→) dp_4 → C → M_1 → P_2 → P_3,M_2 → P^4 (→) M^3 [5]
dc,dp_1,dp_2,dp_3 → dp_4 → C,M_1 → P_2 → P_3,M_2 → P^4 (→) M^3 [3]

Choreopsis liberiensis
Upper jaw: dc → $dp^2\text{-}dp^3$ → dp^1,dp^4 → C,M^1 → M^2 → P^1,P^2,P^3 → P^4 → M^3 [5]
Lower jaw: dc,dp_2,dp_3,dp_4 → C,dp_1,M_1 → P_2,M_2→ P_3 → P^4 (→) M^3 [5]

Here, we describe our observations regarding the upper jaw for the common hippo.

We noticed that the dp^4 is replaced later by the permanent tooth than the dp_4. While the dp^4 was still present, the P_4 was already in place. While the dp_4 has three cusps and is therefore longer than its single-cusped successor, the dp^4 has only two cusps and resembles the molar teeth. In **Fig. S30**, the transition of the deciduous to the permanent dentition of the upper fourth premolar is visible.

The same pattern of an accelerated tooth replacement in the lower jaw could be observed in the eruption pattern of the second and third molar. They usually appear first in the lower jaw and only later in the maxilla. In conclusion, odontogenesis seems to be accelerated in the lower jaw.

Combining molar occlusal area with the dental age categories, it is evident that younger individuals of the common hippo that are still undergoing odontogenesis exhibit an overall smaller molar area, because the second and the third molar tooth erupt with a more advanced age (**Fig. 16**).

Supplementary text: deciduous dentition

The dental formula for the milk teeth in the common hippo is: di 2/2, dc 1/1, dp 4/4. The deciduous first incisors and canines are usually erupted at birth, in contrast to the premolars [3, 4]. The deciduous canines and incisors are similar regarding their dental anatomy, having a peg-like shape and rounded tips. They reach a length of about a centimeter. The canines are shed at around 14 months. Around the time the deciduous canine sheds, the surrounding bone is being suppressed and the permanent tooth erupts slightly behind its predecessor [4]. The first and second incisors are lost at around 1 1/3 and 2 1/2 years of age, respectively.

At around five months the first three deciduous premolars have erupted in both jaws. The fourth premolar follows shortly after, first in the mandible, then in the maxilla (see above). The deciduous premolars, except the first one, are each replaced by a permanent tooth. The first one is not present in every individual and only persists for a varying amount of time [3].

The first premolar usually has only one root, but two may occur [6], and a single cusp. The second deciduous premolar also only has one cusp. The third deciduous mandibular premolar has one main cusp and a pair of smaller cusps behind the former **(Fig. S31)**. In the maxilla, it consists of two single cusps with a pair of cusps following, which results in an increased tooth width in the posterior direction (**Fig. S32**).

In the mandible, the deciduous fourth premolar dp_4 shows three pairs of cusps behind each other, and the outer cusp of the second pair is the most prominent one [4] (**Fig. S33**). The cusp width increases in the posterior cusps (**Fig. S16**), which gives the tooth a slight triangular shape (**Fig. S33**). In the permanent dentition the number of cusps is reduced to one **(Fig. S34)**.

In the maxilla, the deciduous fourth premolar dp^4 consists of one small, sometimes missing, anterior cusp with two pairs of cusps following, the most outstanding one being the antero-external cusp [4] (**Fig. S35**). The permanent P^4 consists of only one cusp **(Fig. S36)**.

The pygmy hippo has a very similar dentition regarding the dental anatomy, but only one pair of the lower milk incisors are replaced by permanent teeth **(Fig. S37-S38)**.

Table S1. Summary of the different age categories correlating with the different teeth patterns in the Hippopotamus amphibius defined by Laws [3]. "Exposed" means protruding above gum level. "Open" means that the tooth is visible in its alveolus, but not protruding above bone level.
D is used for a deciduous tooth, I = Incisor, C = Canine, P = Premolar, M = Molar [7]

Age Class	*Age in Years*		*Mandible Length (mm)*
I	0	D C,I1,I2.P1,P3 exposed. M1 open.	100–160
II	0.5	D C,I1,I2,P1–4 exposed. M1,M2 open.	182–260
III	1	D C,I present. C,I exposed. Wear on D P3,4. M2 open.	274–370
IV	3	D I1,2,3 lost. I1,2 exposed. C,M1 erupted.	318–398
V	4	Wear on C,I,M1. D M1 lost. P2,3,M2 open.	368–424
VI	7	D P1,2 lost. D P4 worn almost flat. M2 exposed.	410–466
VII	8	D P2,3 present. P2,3 erupted. M2 partly erupted. M3 open.	412–496
VIII	11	D M4 present. P2,3 slight wear, P4 exposed, M3 open.	454–516
IX	15	P2–4 erupted. M3 exposed. Wear on M2 on 2 cusps.	472–530
X	17	M3 erupted. Dentine on M1 usually continuous from wear.	462–536
XI	20	M1 dentine continuous. Slight wear on M3. Wear on P2,4.	496–544
XII	22	Increased wear P2–4. M1 worn almost flat.	496–588
XIII	24	M1 worn flat. M3 dentine exposed on 1st & 2nd cusps.	478–550
XIV	27	Further wear on all teeth. M2 cusps not yet joined.	482–570
XV	30	M1 flat/concave. 50% M2 cusps joined. 3rd cusp M3 worn.	506–566
XVI	33	M2 dentine continuous. Further wear on M3.	490–588
XVII	35	P2–4,M2 worn flat. M1 worn to gum. Continuous dentine M3.	510–602
XVIII	38	M1–3 worn flat.	510–574
XIX	40	M1 below gum, M2 concave, M3 flat. Resorption of bone.	490–556
XX	43	Usually only P3,4,M2,3 still present and very worn.	498–528

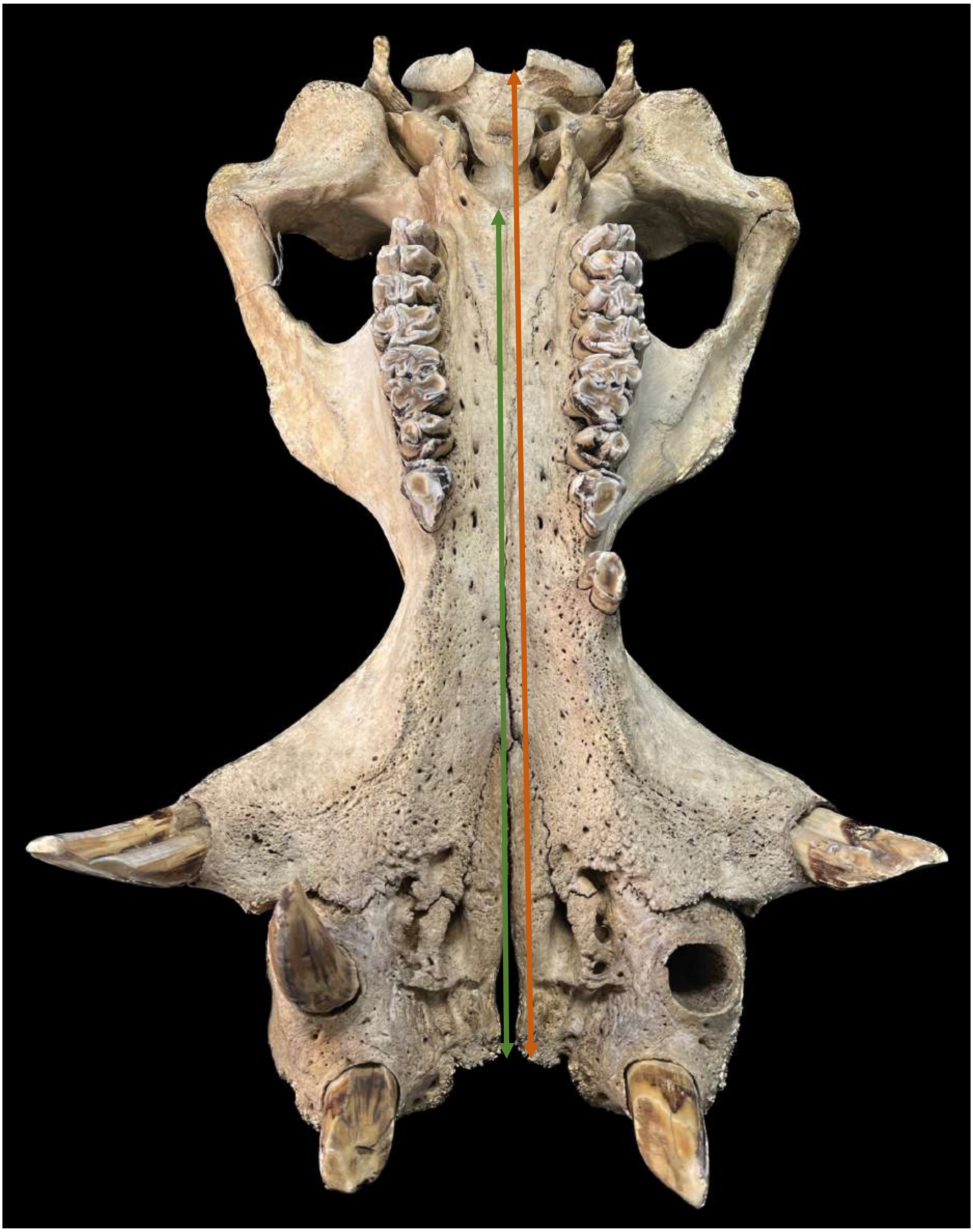

Figure S1 Ventrodorsal view of an upper skull of an adult common hippo (*Hippopotamus amphibius*). The orange arrow marks the measured distance between the *Processus palatinus ossis incisivi* and the *Os occipitale*, the green arrow illustrates the measured distance between the *Processus palatinus ossis incisivi* and the *Os palatinum*. Photo: Annika Avedik.

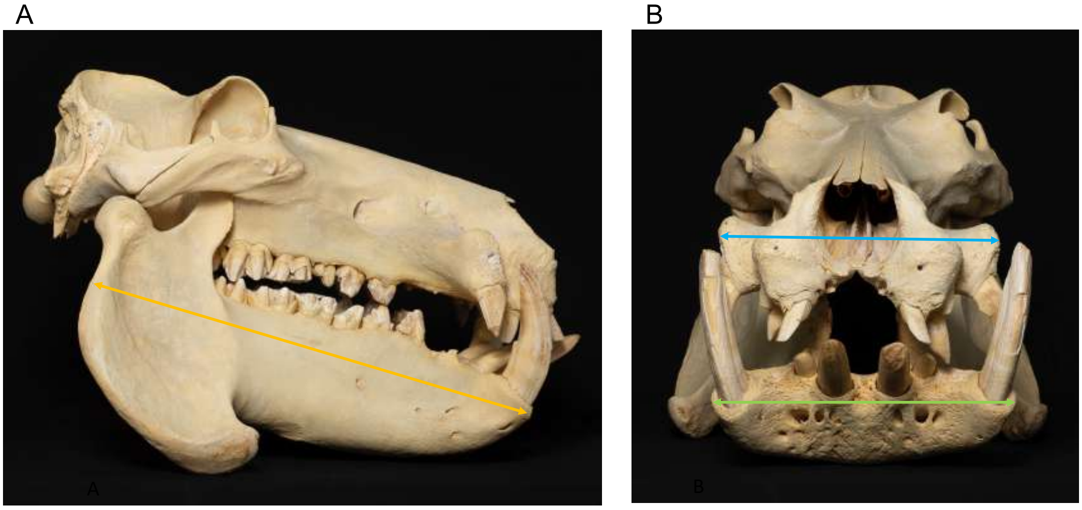

Figure S2 (A) Lateral view of an adult common hippo (*Hippopotamus amphibius*) skull. We determined the length of the mandible by measuring the length between the *Ramus mandibulae* and the cranial margin of the *Corpus mandibulae* (yellow arrow). **(B)** Frontal view of an adult *Hippopotamus amphibius* skull. We measured the distance between the upper (blue arrow) and lower canines (green arrow). Photos: Michelle Aimée Oesch.

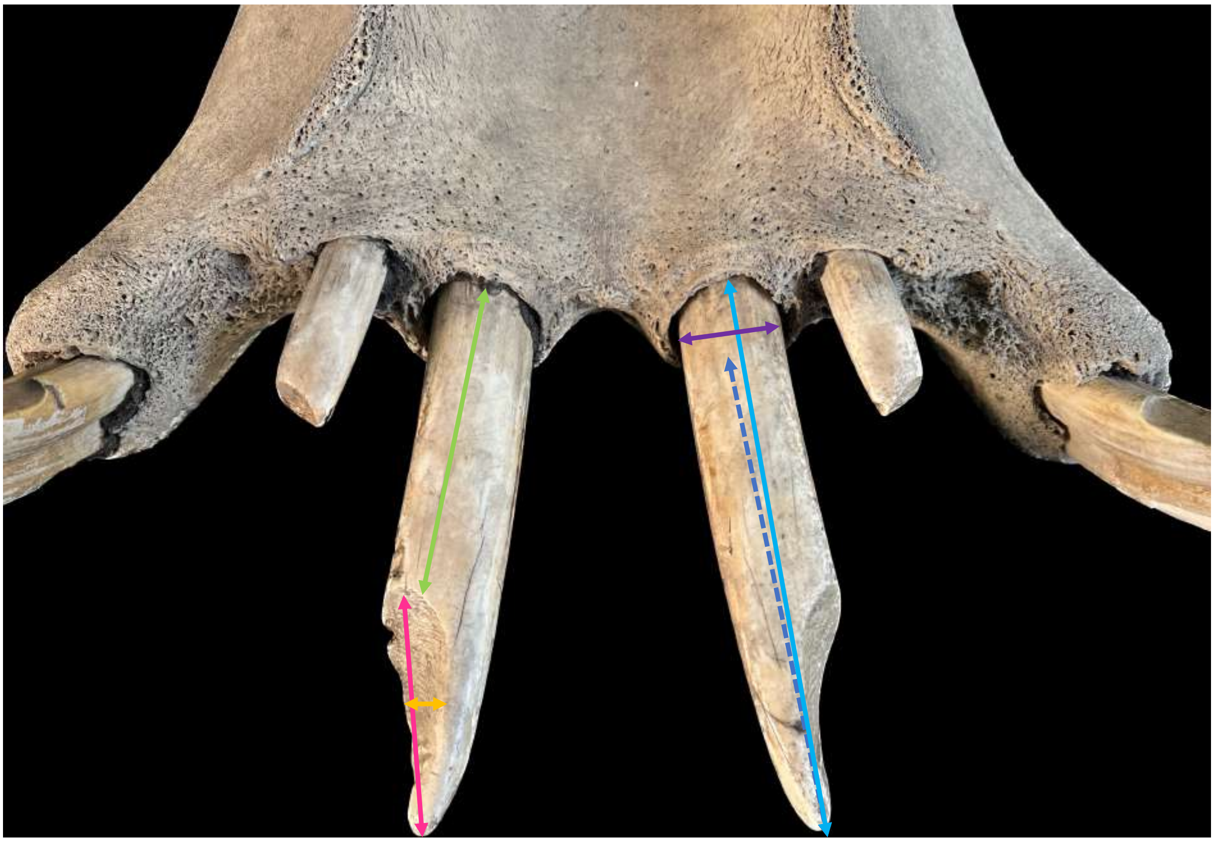

Figure S3 Dorsal view of the lower incisors of a common hippo (*Hippopotamus amphibius*). We measured the length from the tooth alveolus to the tip of the tooth on the lingual (light blue arrow) and the mesial (dashed dark blue arrow) side of the tooth. The width of the incisors was determined at the base of the tooth (purple arrow). We determined the length between the tooth alveolus and the wear facet (light green arrow). The length (pink arrow) and the width (yellow arrow) of the wear facet was measured, as well as the distances between the incisors. Photo: Annika Avedik.

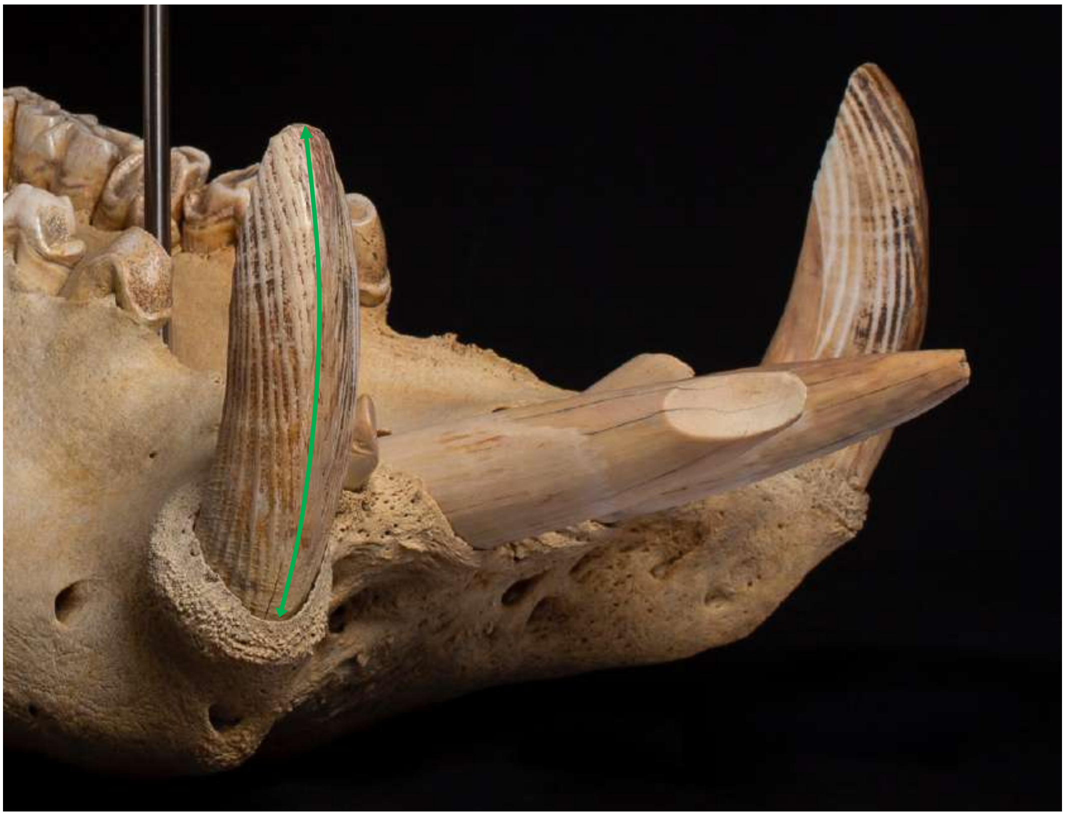

Figure S4 Craniolateral view of the anterior part of a mandible of a common hippo (*Hippopotamus amphibius*). The mesial tooth length of the canines was determined (bright green arrow). Photo: Michelle Aimée Oesch.

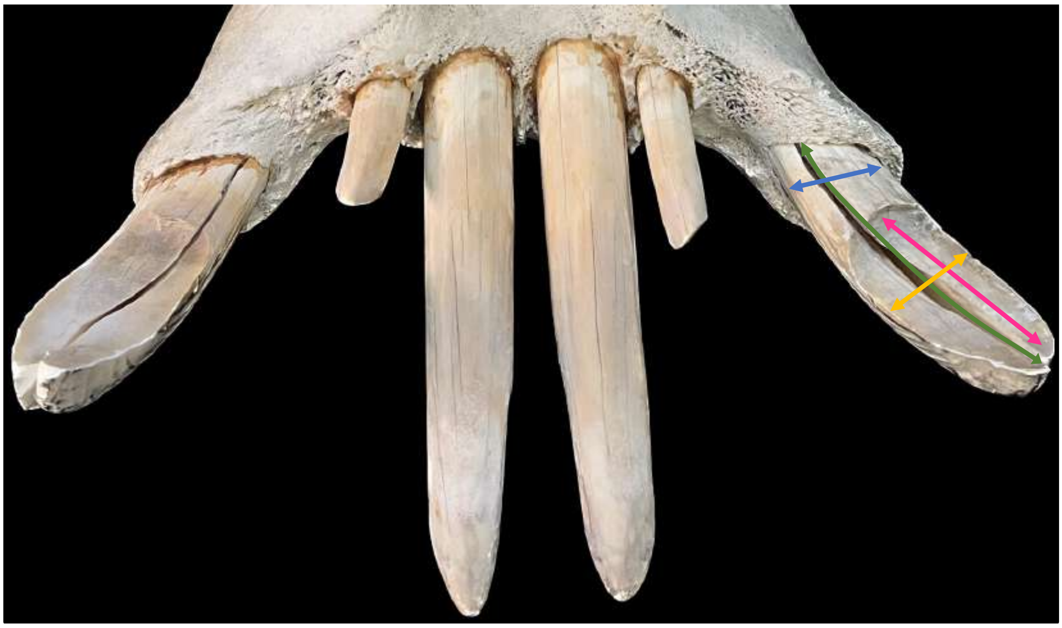

Figure S5 Dorsoventral view of the lower anterior dentition of a common hippo (*Hippopotamus amphibius*). We measured the basal tooth width (dark blue arrow), the lingual tooth length (dark green arrow), the facet width (yellow arrow) and length (pink arrow). Photo: Annika Avedik.

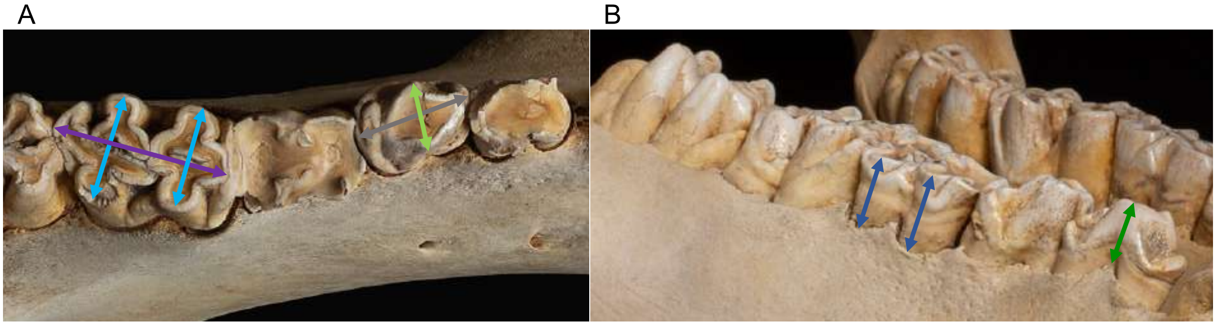

Figure S6. (A) Dorsoventral view of the lower posterior dentition of a common hippo (*Hippopotamus amphibius*). The tooth length of the premolar and molar teeth (grey/violet arrow) was determined in anterioposterior direction on the central axis of the tooth. We measured the width (light green/light blue arrow) of every cusp in the premolar or cusp-pair in the molar teeth. **(B)** Lateral view of the posterior dentition of a common hippo (*Hippopotamus amphibius*). The height was measured at the buccal side of the cusp (dark blue/green arrow). Photos: Michelle Aimée Oesch.

1 Crypt

2 Tooth visible in crypt, but below bone

3 Tooth erupting through bone

4 Tooth half erupted

5 Tooth fully erupted but fully unworn (no dentine exposed and no wear facets on enamel

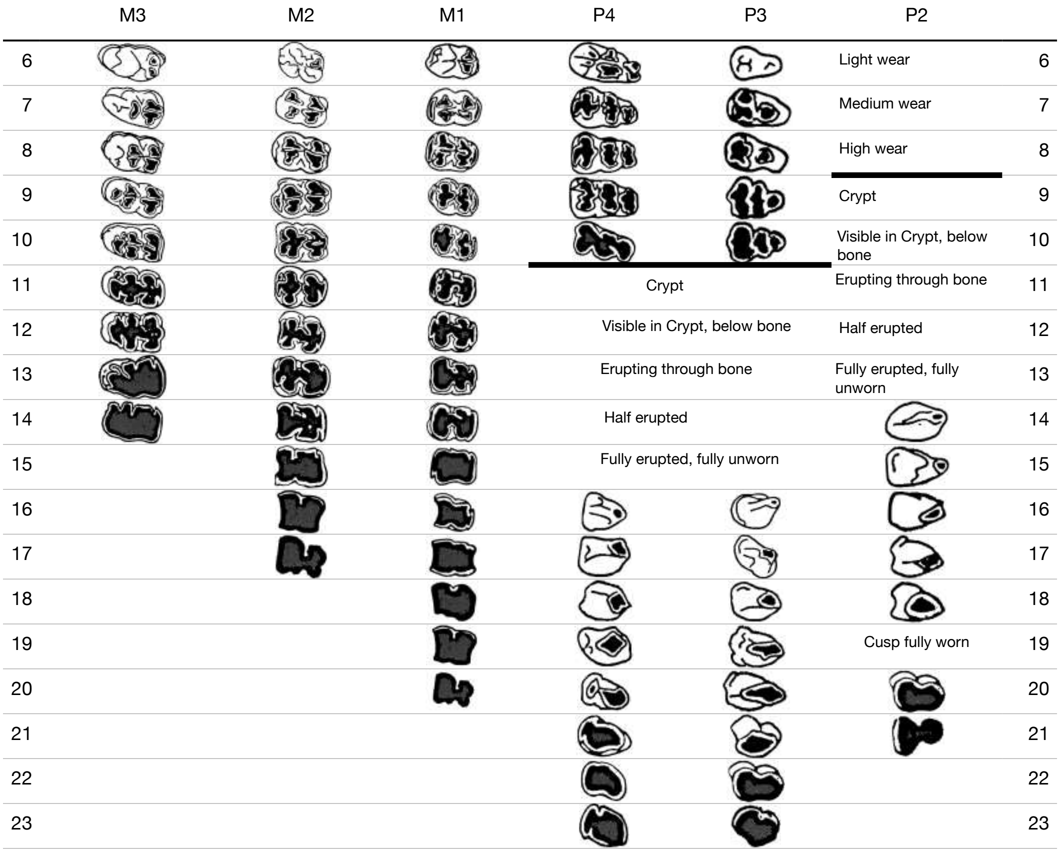

Figure S7. Macrowear scoring table designed to quantify tooth wear of the premolar and molar teeth in the common hippo (*Hippopotamus amphibius*). Based on the drawings of Laws [3], with some modified or added wear stages.

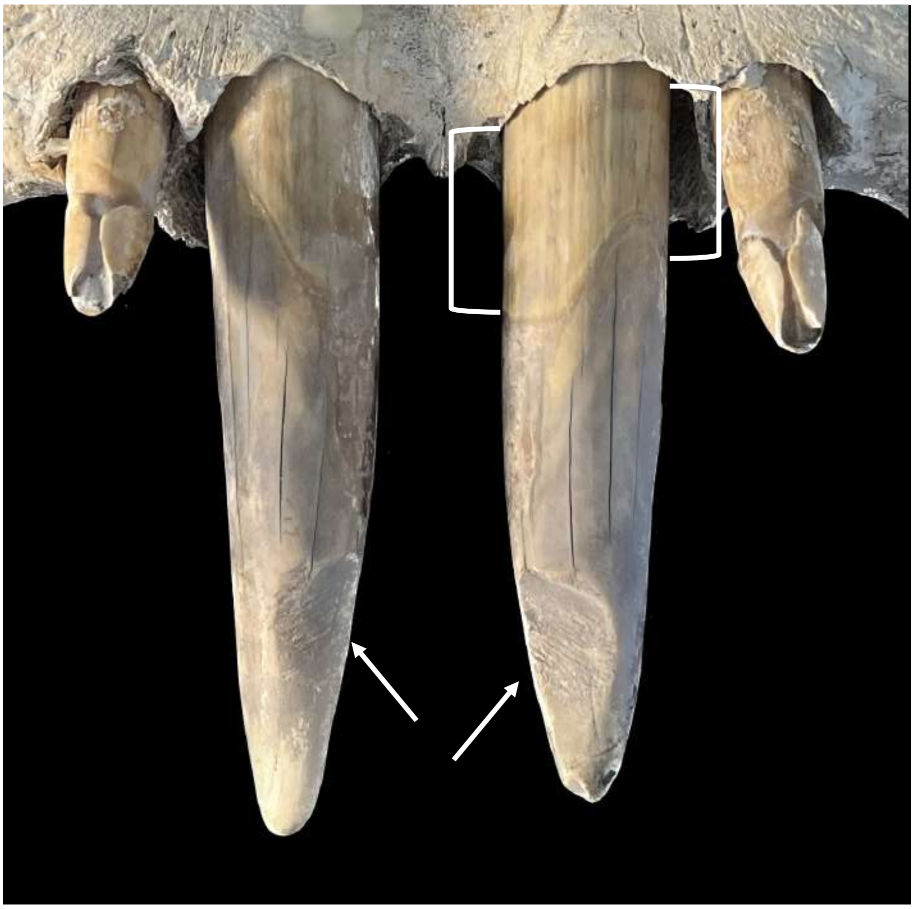

Figure S8 Dorsoventral view of the lower incisors of a common hippo (*Hippopotamus amphibius*). The shading on the first incisors does not match the rim of the alveolar bone (difference marked by the brackets), possibly indicating false museal positioning of the incisors in their alveolar socket. The arrows indicate incisor wear facets facing the wrong direction, also indicating museal mispositioning of the teeth. Photo: Annika Avedik.

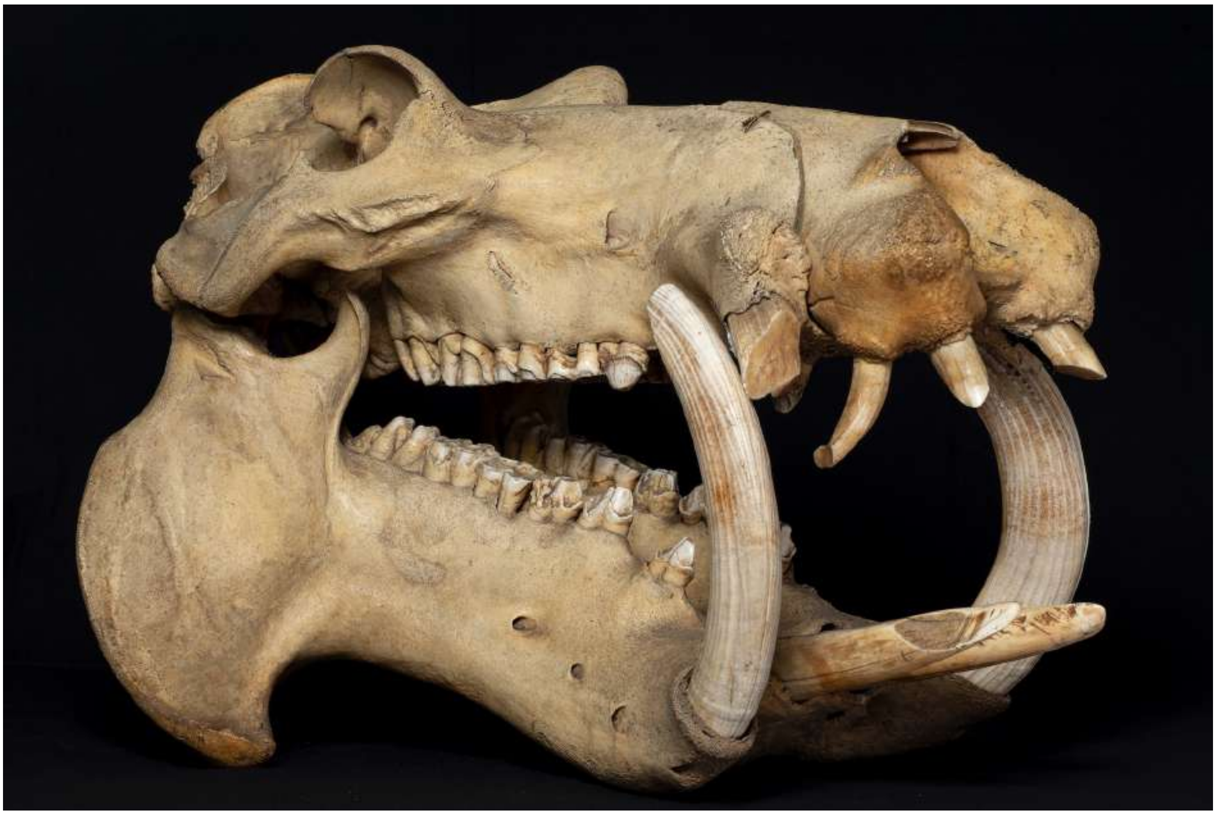

Figure S9 Fronto-lateral view of a semi-opened skull of a common hippo (*Hippopotamus amphibius*). The canines are not fully positioned in their alveoli (possibly to create a more impressive display), preventing the closing of the skull. Note the abnormally growing right I^2, and the typical wear facets on the I^1 and I_1. Photo: Michelle Aimée Oesch.

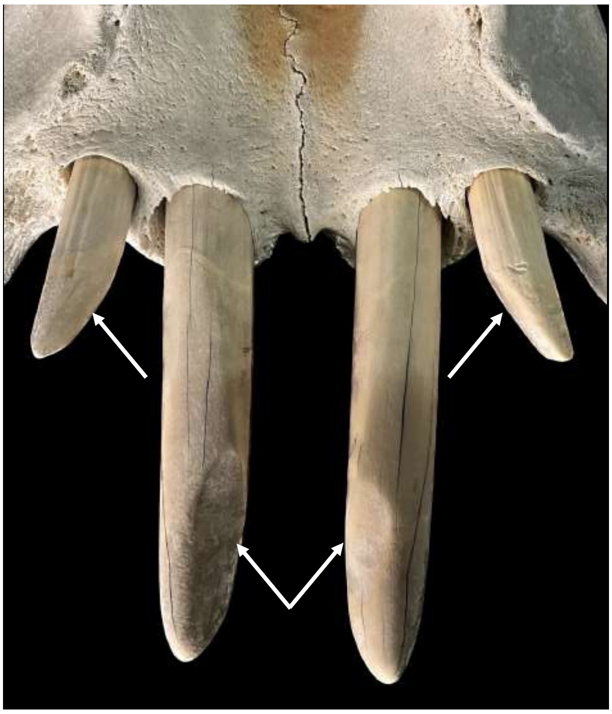

Figure S10 Dorsoventral view of the lower incisors of a common hippo (*Hippopotamus amphibius*). The wear facets of both lower incisor pairs are facing the wrong direction due to museal mispositioning. Photo: Annika Avedik.

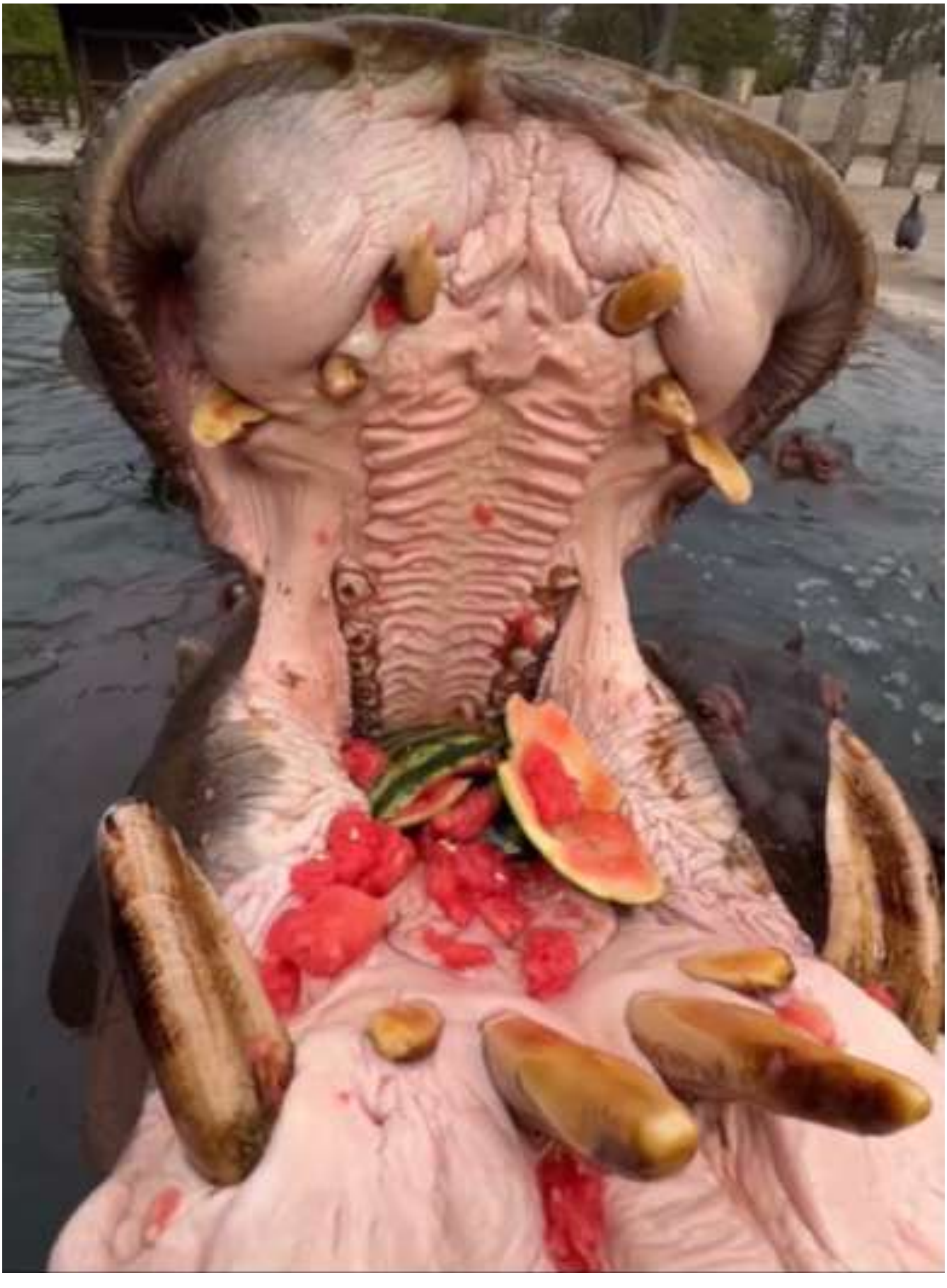

Figure S11 Frontal view of live of a common hippo (*Hippopotamus amphibius*), showing the position of the wear facets of the lower and upper incisors. Photo: Brian Stefanski.

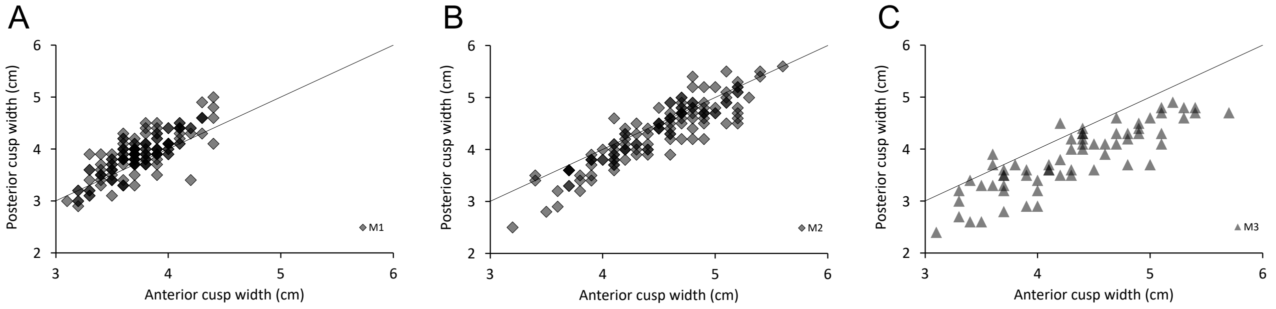

Figure S12 Comparison of cusp width in the common hippo (*Hippopotamus amphibius*) maxillary molars. **(A)** M1, **(B)** M2, **(C)** M3. The line denotes y = x. Darker shading indicates overlaying data points.

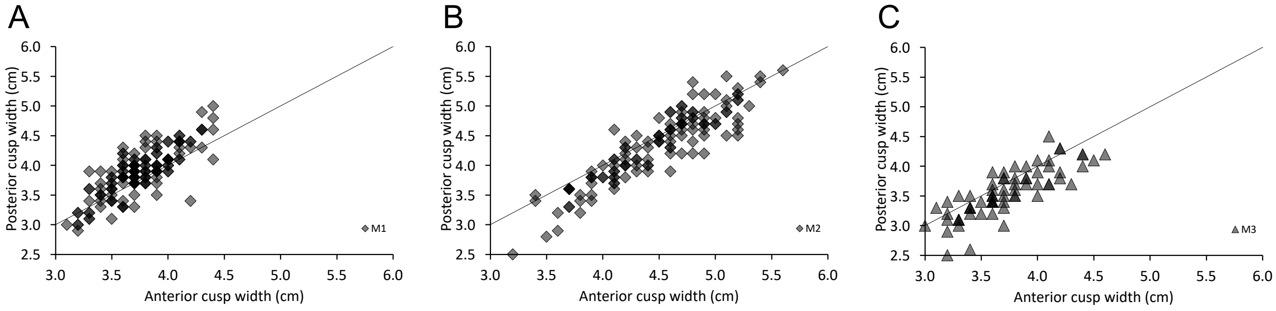

Figure S13 Comparison of cusp width in the common hippo (*Hippopotamus amphibius*) mandibular molars. **(A)** M1, **(B)** M2, **(C)** M3. The line denotes y = x. Darker shading indicates overlaying data points.

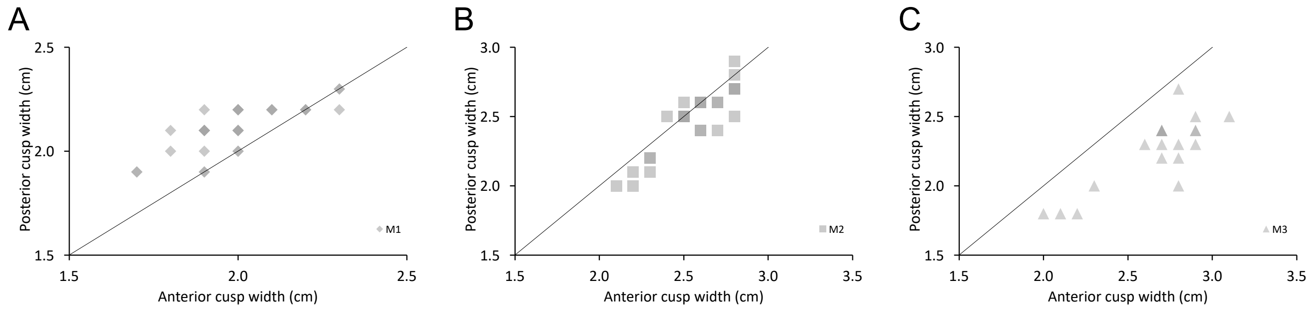

Figure S14 Comparison of cusp width in the pygmy hippo (*Choeropsis liberiensis*) maxillary molars. **(A)** M1, **(B)** M2, **(C)** M3. The line denotes y = x. Darker shading indicates overlaying data points.

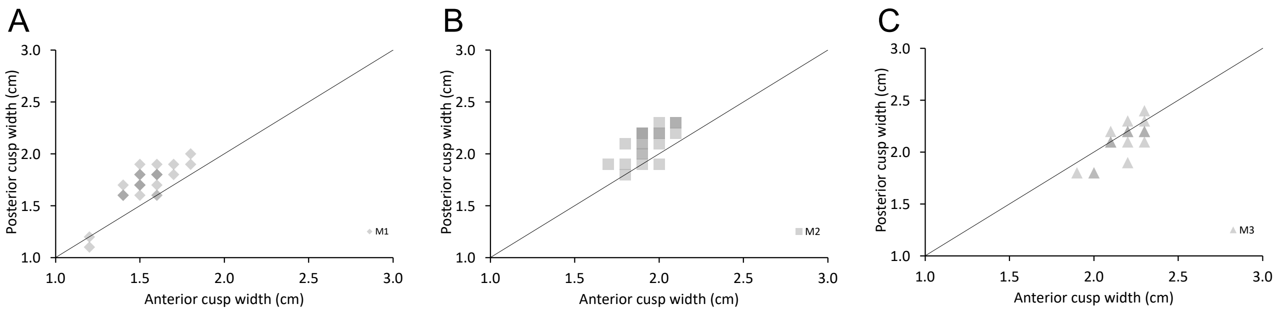

Figure S15 Comparison of cusp width in the pygmy hippo (*Choeropsis liberiensis*) mandibular molars. **(A)** M1, **(B)** M2, **(C)** M3. The line denotes y = x. Darker shading indicates overlaying data points.

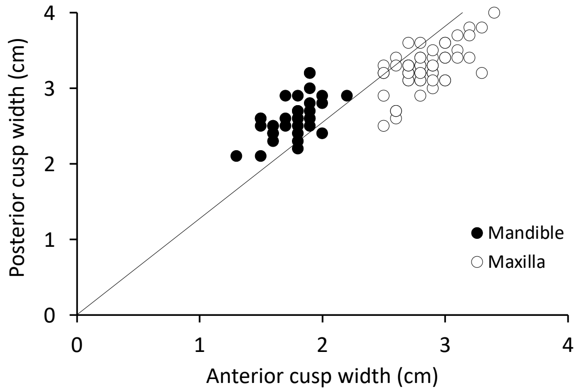

Figure S16 Comparison of cusp width in the common hippo (*Hippopotamus amphibius*) maxillary and mandibular deciduous p4. The line denotes y = x.

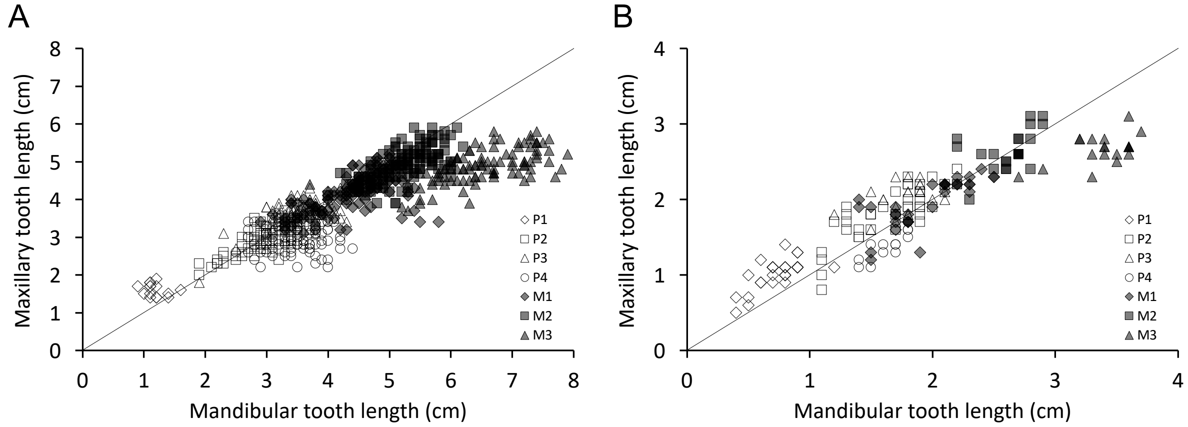

Figure S17 Comparison of maxillary and mandibular tooth length in hippos: **(A)** common hippo (*Hippopotamus amphibius*), **(B)** pygmy hippo (*Choeropsis liberiensis*). The line denotes y = x.

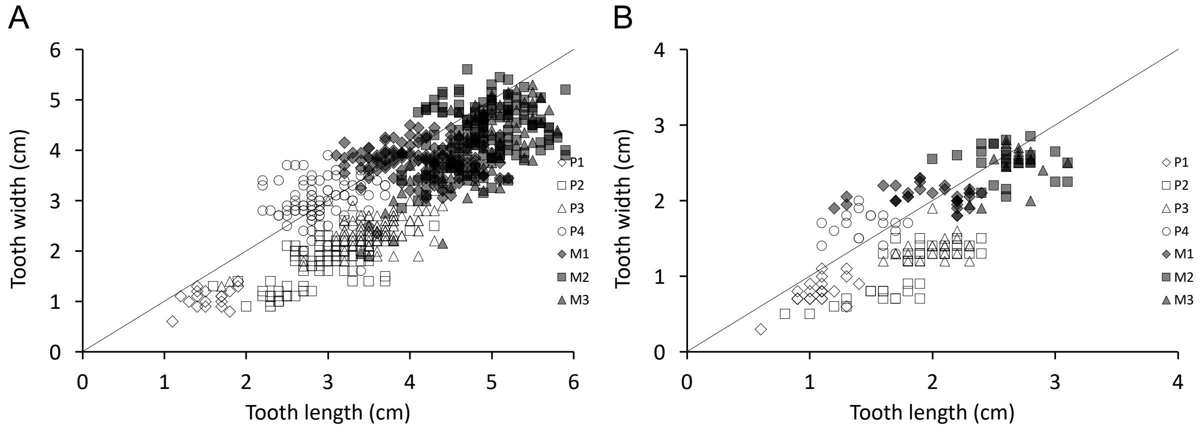

Figure S18 Comparison of tooth length and width of maxillary premolars and molars in hippos: **(A)** common hippo (*Hippopotamus amphibius*), **(B)** pygmy hippo (*Choeropsis liberiensis*). The line denotes y = x.

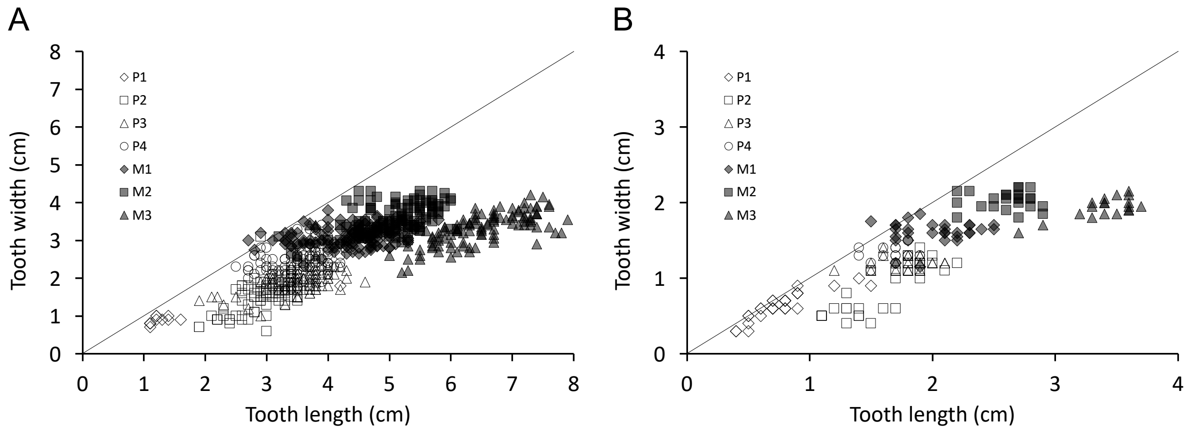

Figure S19 Comparison of tooth length and width of mandibular premolars and molars in hippos: **(A)** common hippo (*Hippopotamus amphibius*), **(B)** pygmy hippo (*Choeropsis liberiensis*). The line denotes y = x.

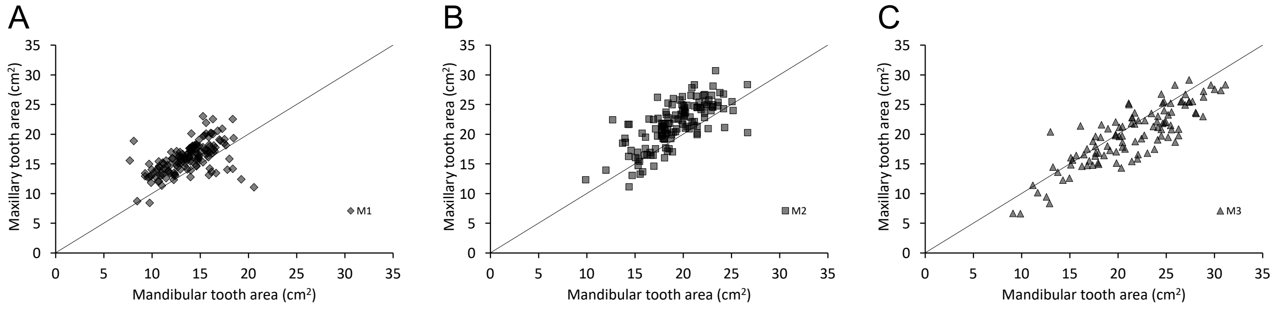

Figure S20 Comparison of the maxillary and mandibular molar tooth area in the common hippo (*Hippopotamus amphibius*). **(A)** M1, **(B)** M2, **(C)** M3. The line denotes y = x. Darker shading indicates overlaying data points.

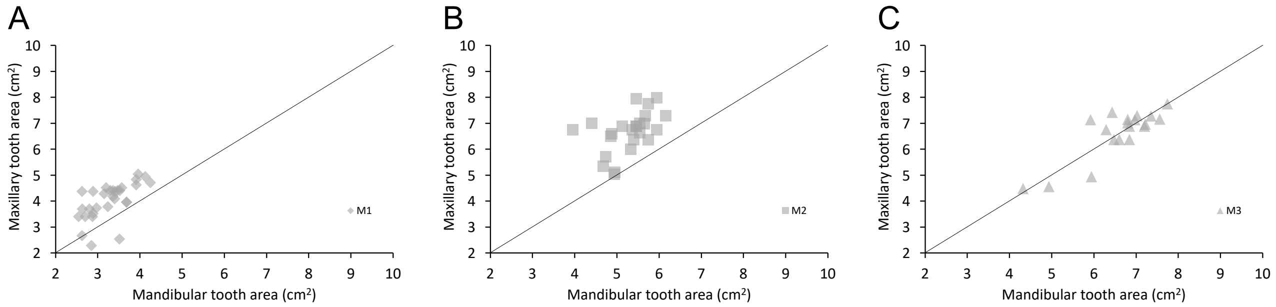

Figure S21 Comparison of the maxillary and mandibular molar tooth area the pygmy hippo (*Choeropsis liberiensis*). **(A)** M1, **(B)** M2, **(C)** M3. The line denotes y = x. Darker shading indicates overlaying data points.

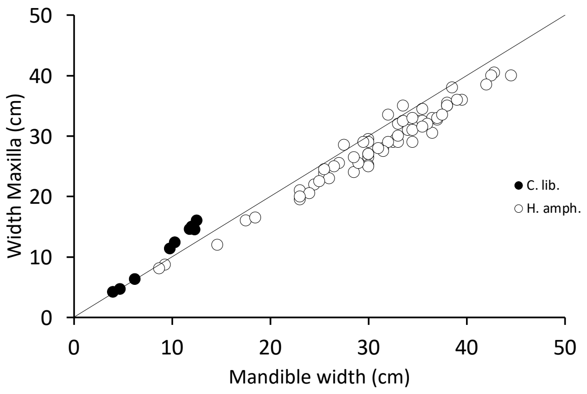

Figure S22 Comparison of the mandible and maxilla width, measured at the level of the canines in hippos (*Hippopotamus amphibius*, *Choeropsis liberiensis*). The line denotes y = x.

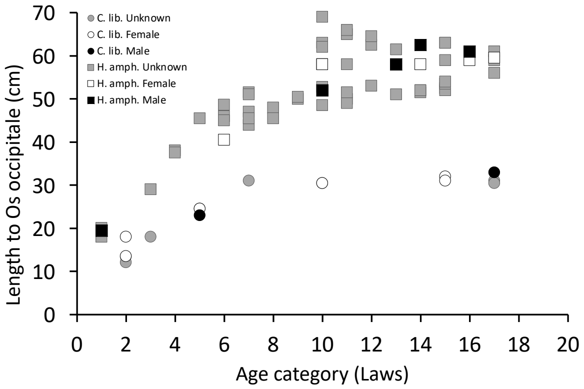

Figure S23 Comparison of the upper skull length and the age category from Laws [3] in hippos (*Hippopotamus amphibius*, *Choeropsis liberiensis*).

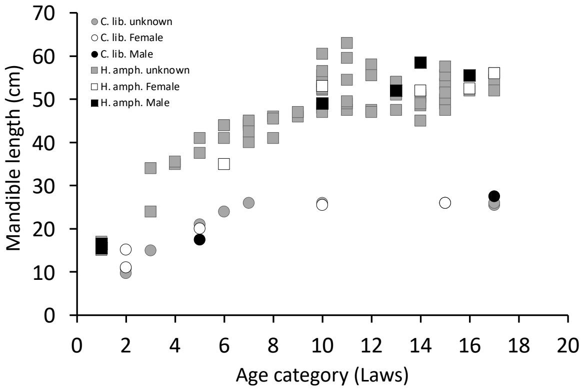

Figure S24 Comparison of the mandible length and the age category from Laws [3] in hippos (*Hippopotamus amphibius*, *Choeropsis liberiensis*).

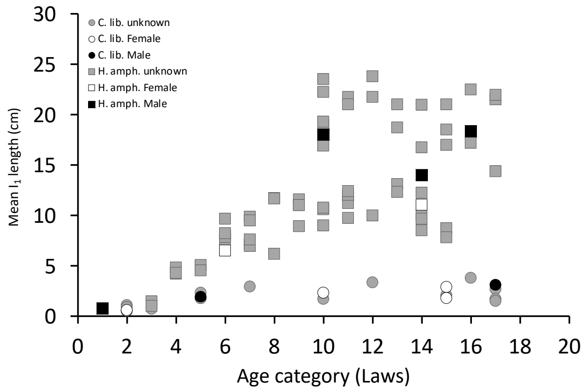

Figure S25 Comparison of the length of the I_1 and the age category from Laws [3] in hippos (*Hippopotamus amphibius*, *Choeropsis liberiensis*).

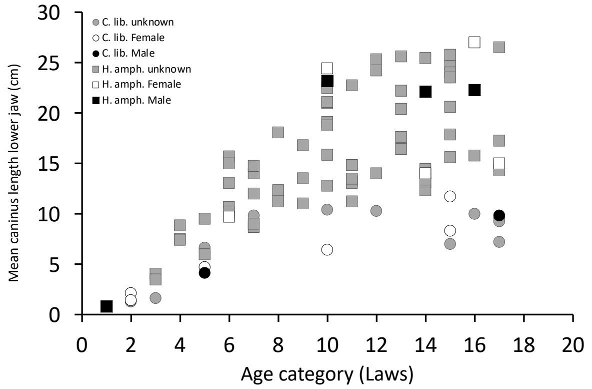

Figure S26 Comparison of the canine length and the age category from Laws [3] in hippos (*Hippopotamus amphibius*, *Choeropsis liberiensis*).

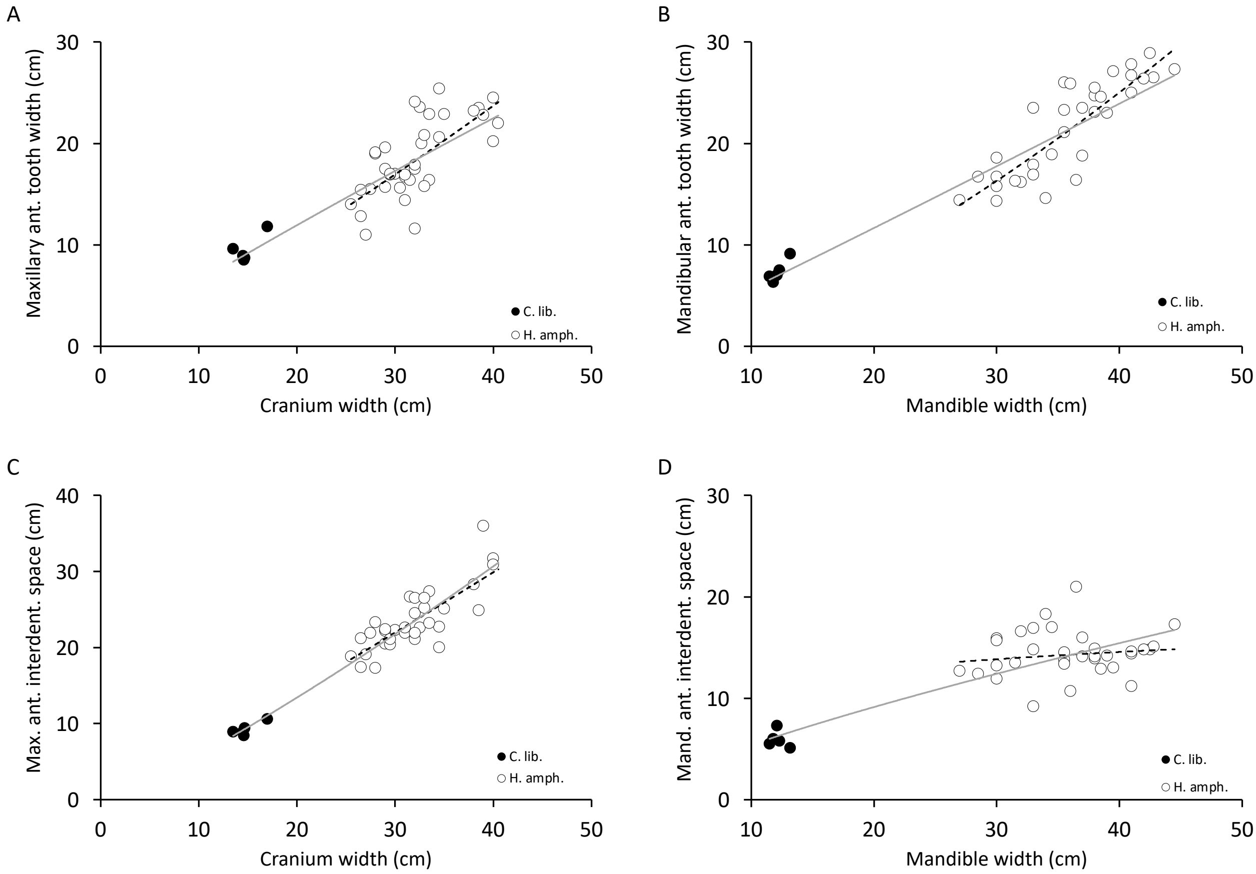

Figure S27 Scaling relationships of the width of the (A) maxillary and (B) mandibular anterior teeth row (I,C) or the interdental space between them for the (C) cranium and (D) the mandible in adult common hippo (*Hippopotamus amphibius*) and pygmy hippo (*Choeropsis liberiensis*). The scaling exponents (with 95%CI) of the grey line (across all individuals of both species) are (A) 0.91 (0.75,1.08), (B) 1.04 (0.93,1.15), (C) 1.19 (1.09,1.30), (D) 0.76 (0.61,0.90); of the dotted line (within *H. amphibius*) (A) 1.17 (0.79,1.35), (B) 1.49 (1.16,1.82), (C) 1.07 (0.79,1.35), (D) 0.18 (-0.25,0.60). If the 95%CI of the scaling exponent is < 1, this indicates negative allometry, if it is > 1, it indicates positive allometry.

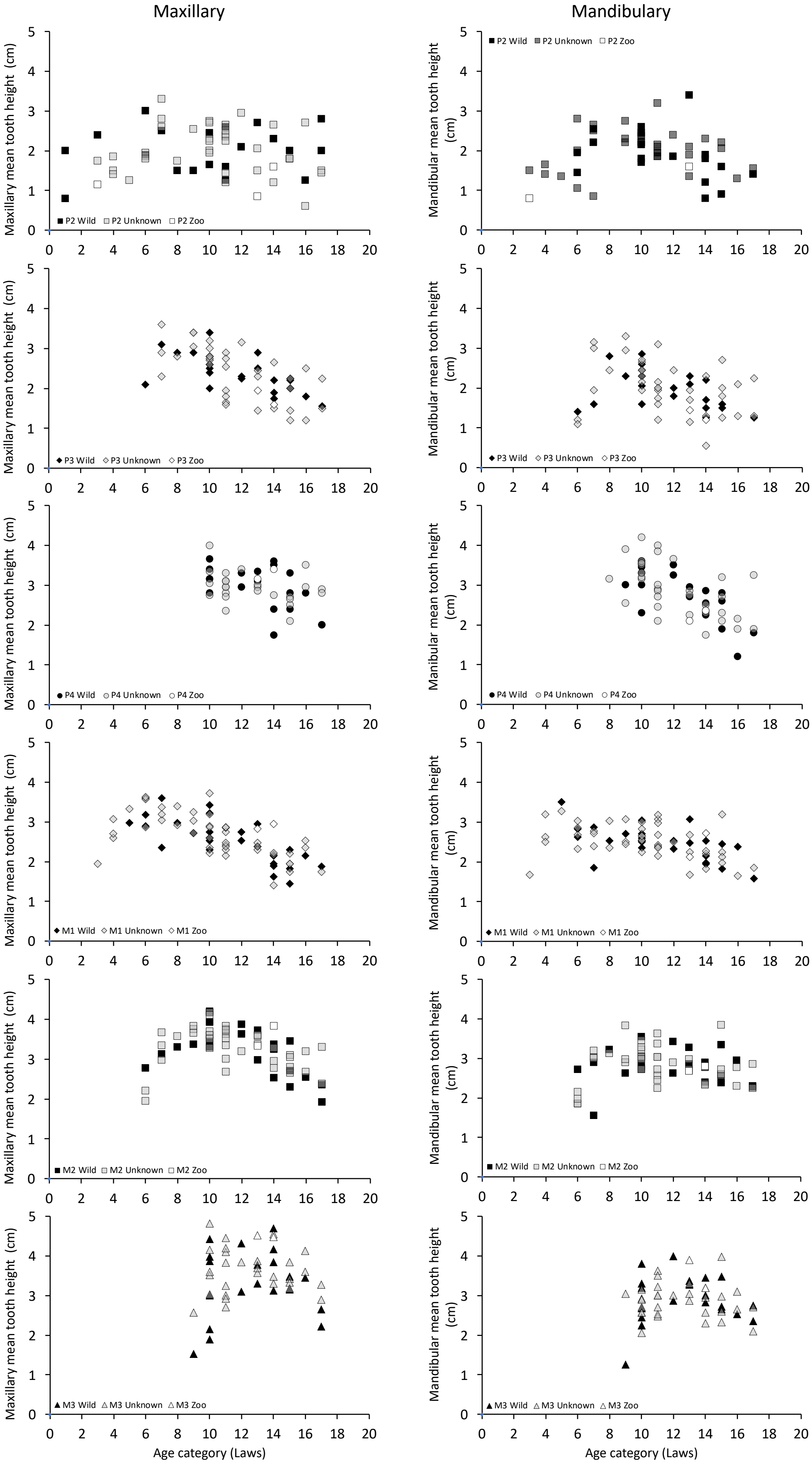

Figure S28 Tooth height in relation to the estimated age class in common hippo (*Hippopotamus amphibius*) of known (wild, zoo) or unknown origin.

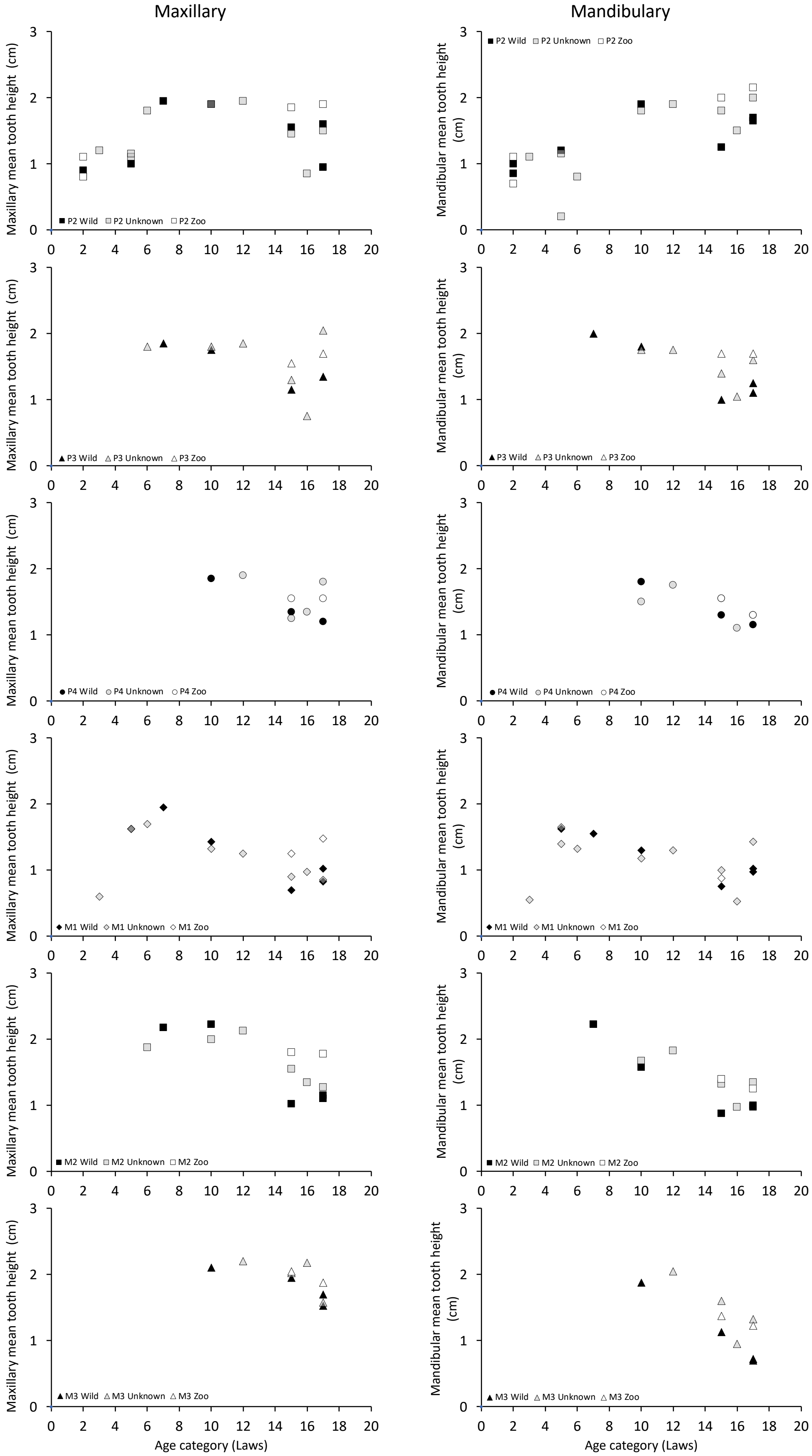

Figure S29 Tooth height in relation to the estimated age class in pygmy hippo (*Choeropsis liberiensis*) of known (wild, zoo) or unknown origin.

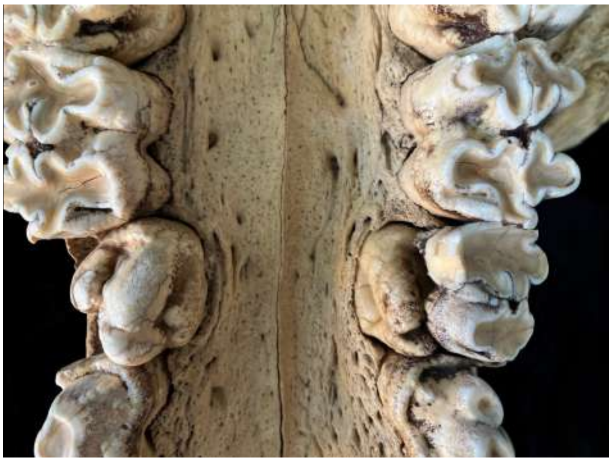

Figure S30 Deciduous tooth replacement of the upper fourth premolar in a common hippo (*Hippopotamus amphibius*). On the left side, the single-cusped permanent P^4 is visible, while on the right side the permanent P^4 has erupted halfway, while the p^4 is still present. Photo: Annika Avedik.

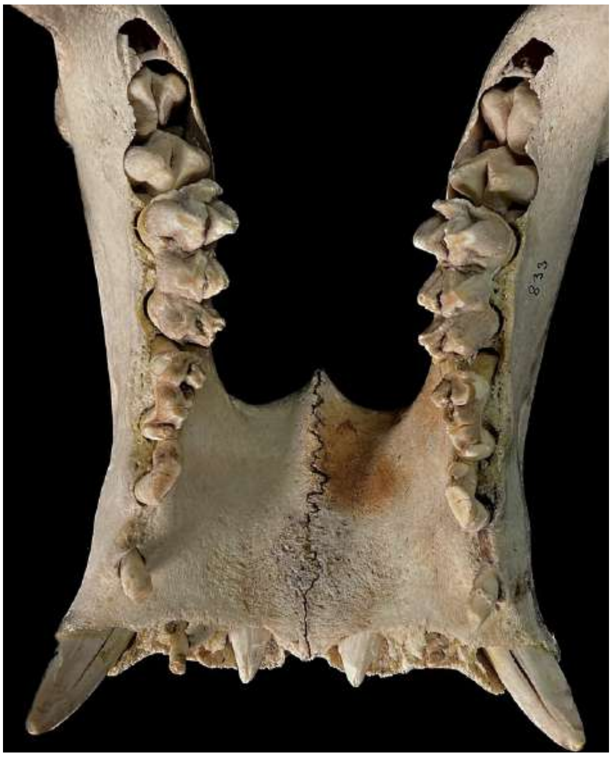

Figure S31 The mandible of a juvenile *Hippopotamus amphibius*. The deciduous second incisors and first three premolars are present, while the first incisors and canines have already been replaced by the permanent teeth. On both sides, the first molars are protruding just above the bone level. Photo: Annika Avedik.

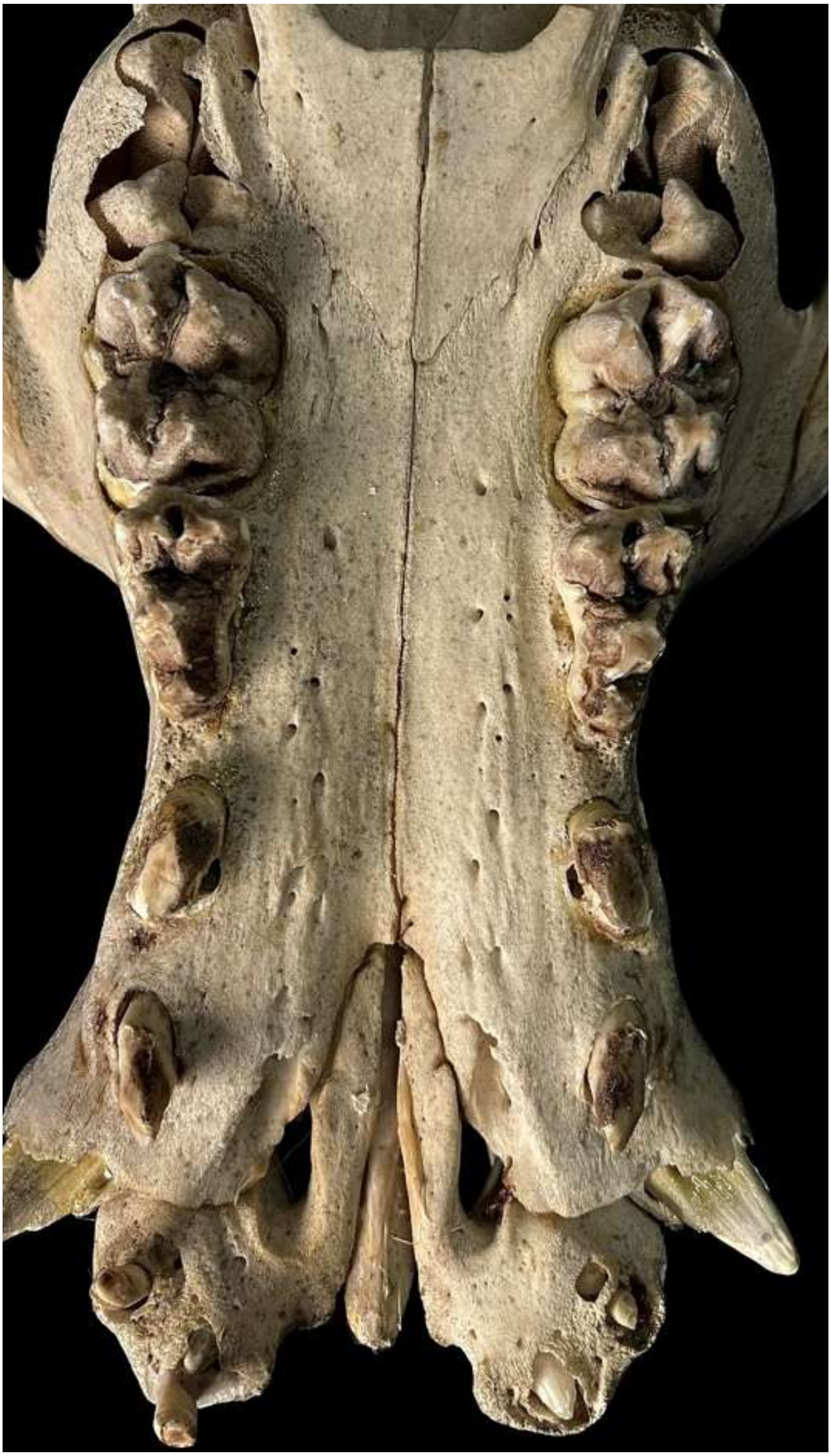

Figure S32 Maxilla of a juvenile *Hippopotamus amphibius*. The milk dentition of the left incisors and first four premolars is visible. The incisors on the right side and both the canines are already replaced by the permanent dentition. The first molars are visible below the bone level, not yet protruding. Photo: Annika Avedik.

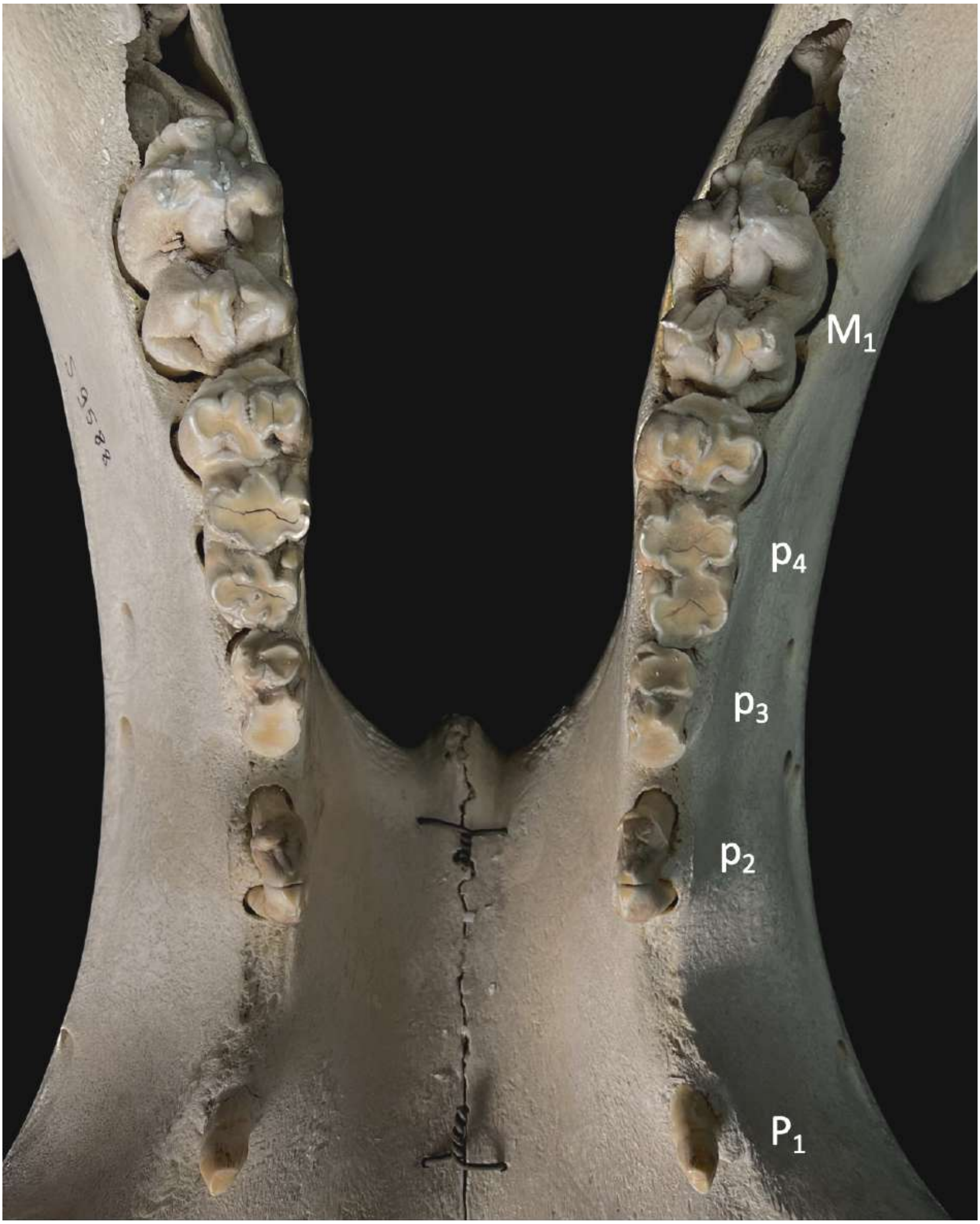

Figure S33 Mandible of a juvenile *Hippopotamus amphibius,* with a partial milk dentition. Photo: Annika Avedik.

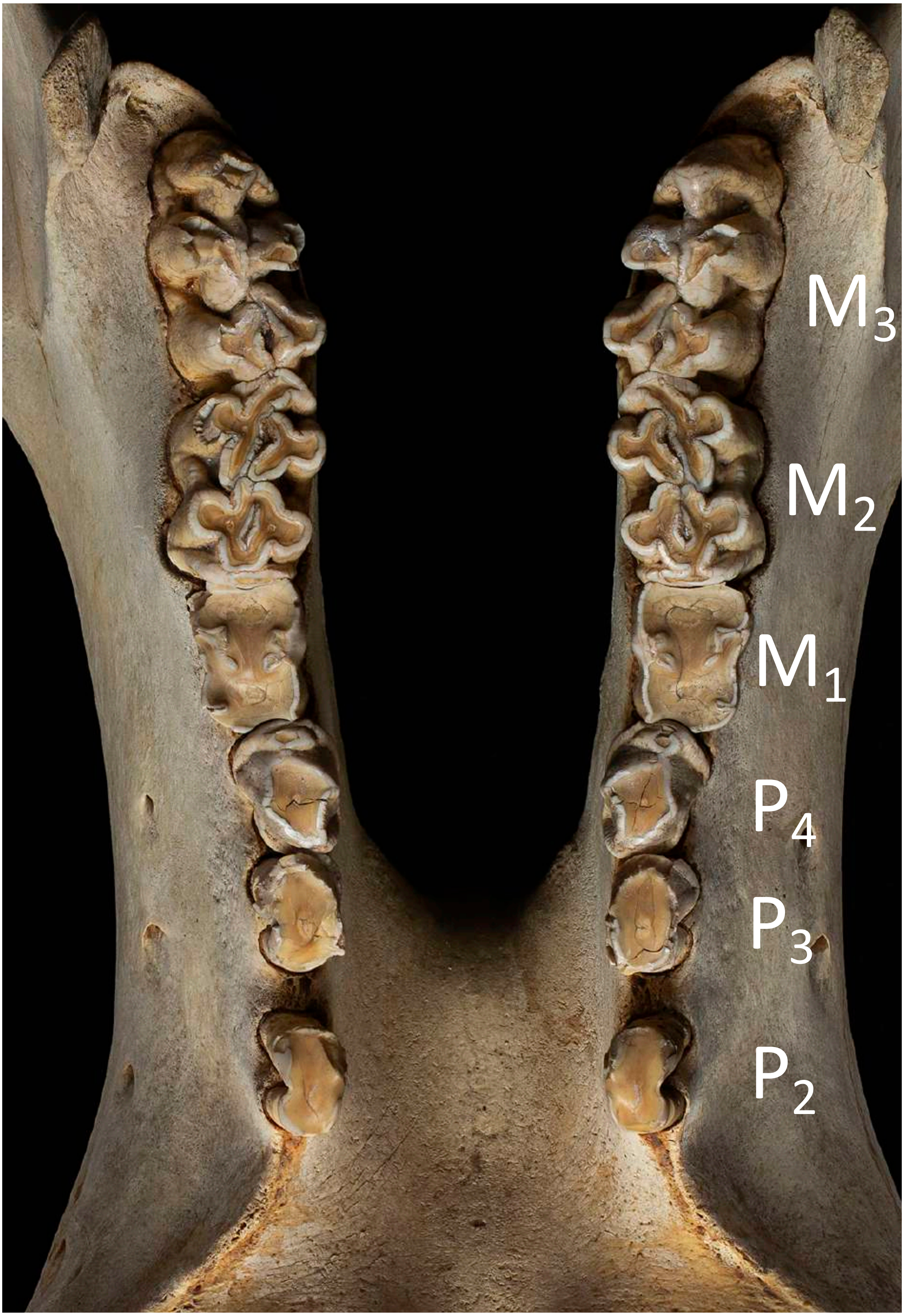

Figure S34 Mandible of an adult *Hippopotamus amphibius*. Photo: Michelle Aimée Oesch.

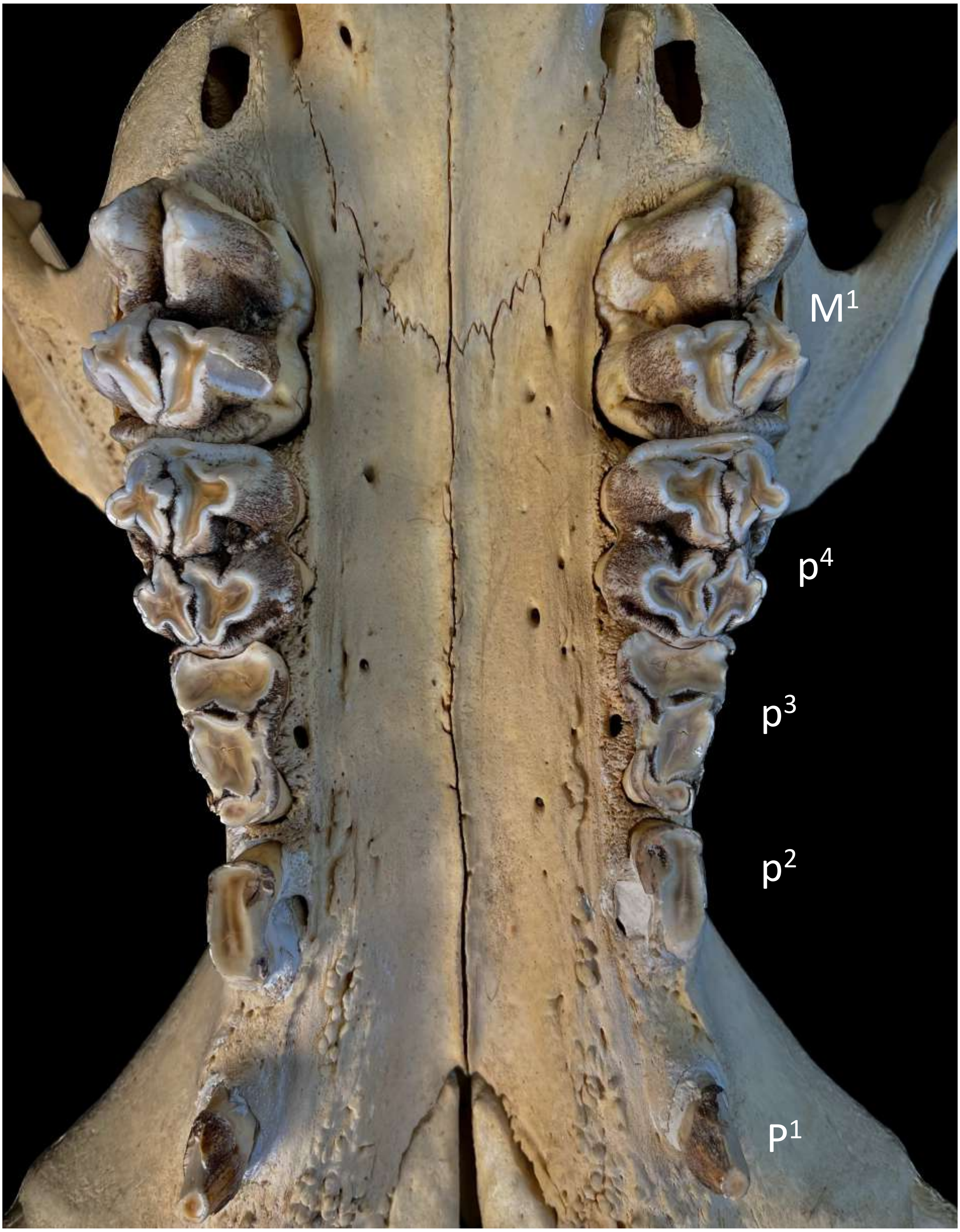

Figure S35 The upper jaw of a juvenile *Hippopotamus amphibius.* The two-cusped p^4 is visible. Photo: Annika Avedik.

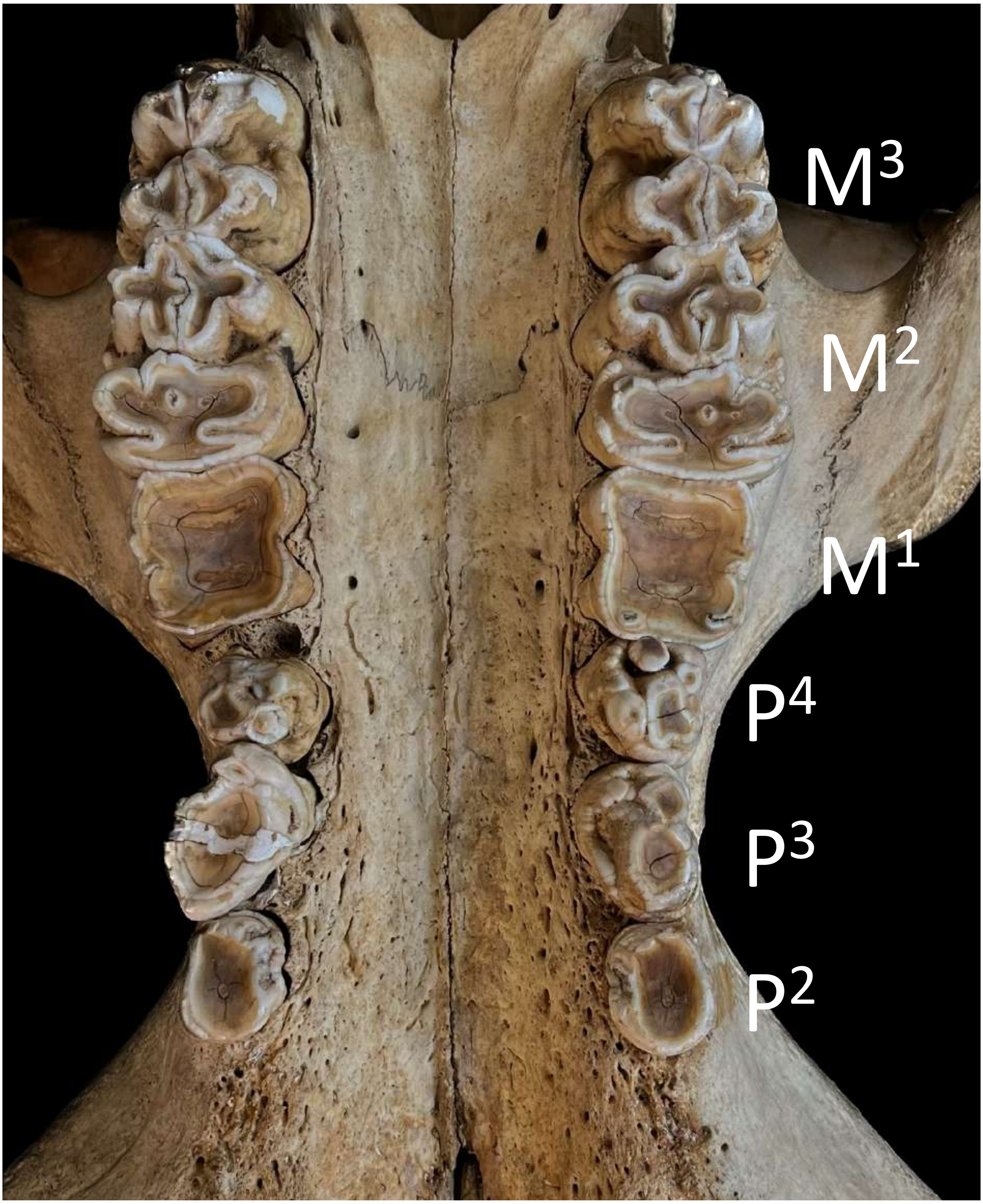

Figure S36 The upper jaw of an adult common *Hippopotamus amphibius.* The P^4 with only one cusp is visible. Photo: Annika Avedik.

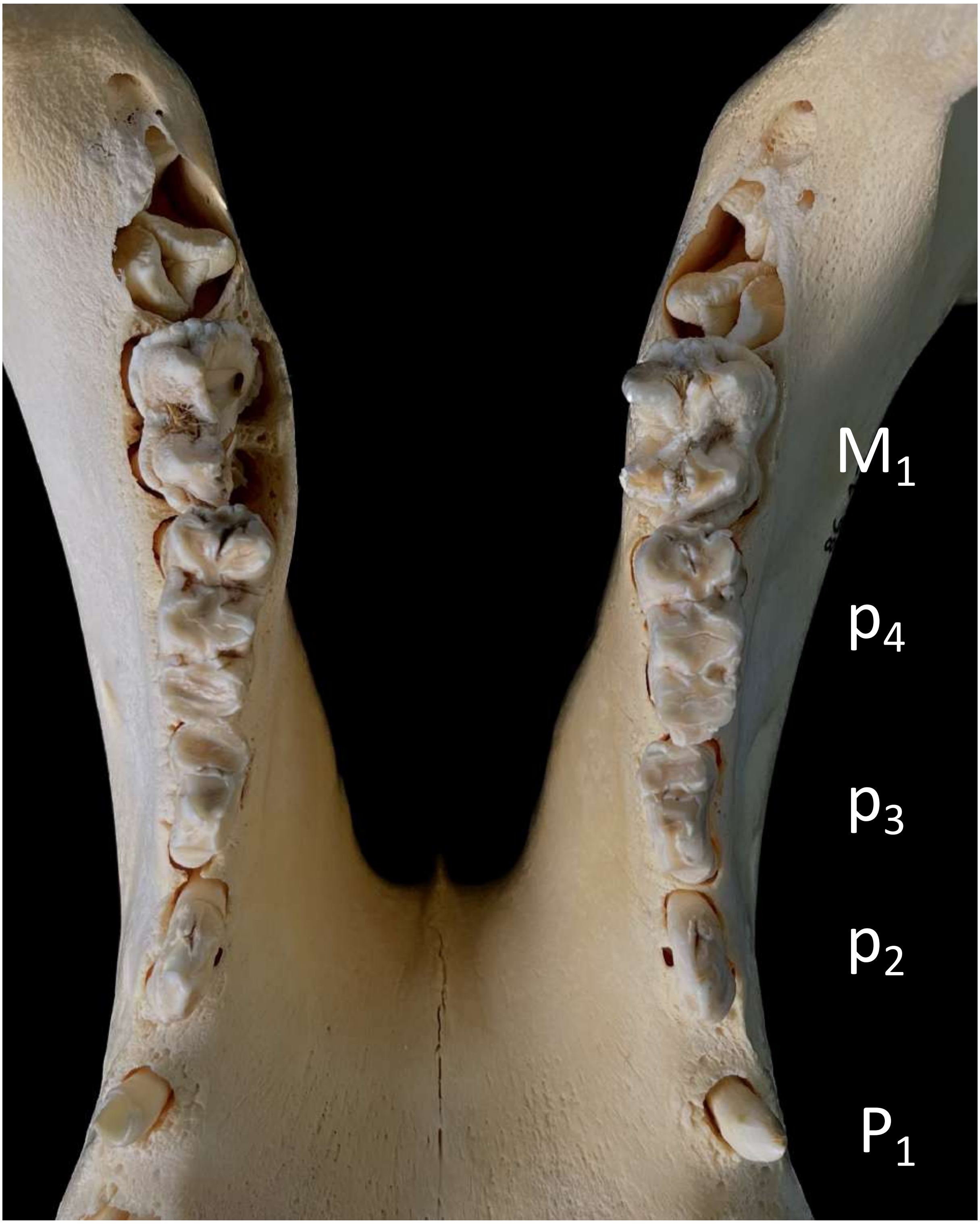

Figure S37 Mandibular milk dentition of a pygmy hippo (*Choeropsis liberiensis).* Half of the right M_1 has broken off. Photo: Annika Avedik.

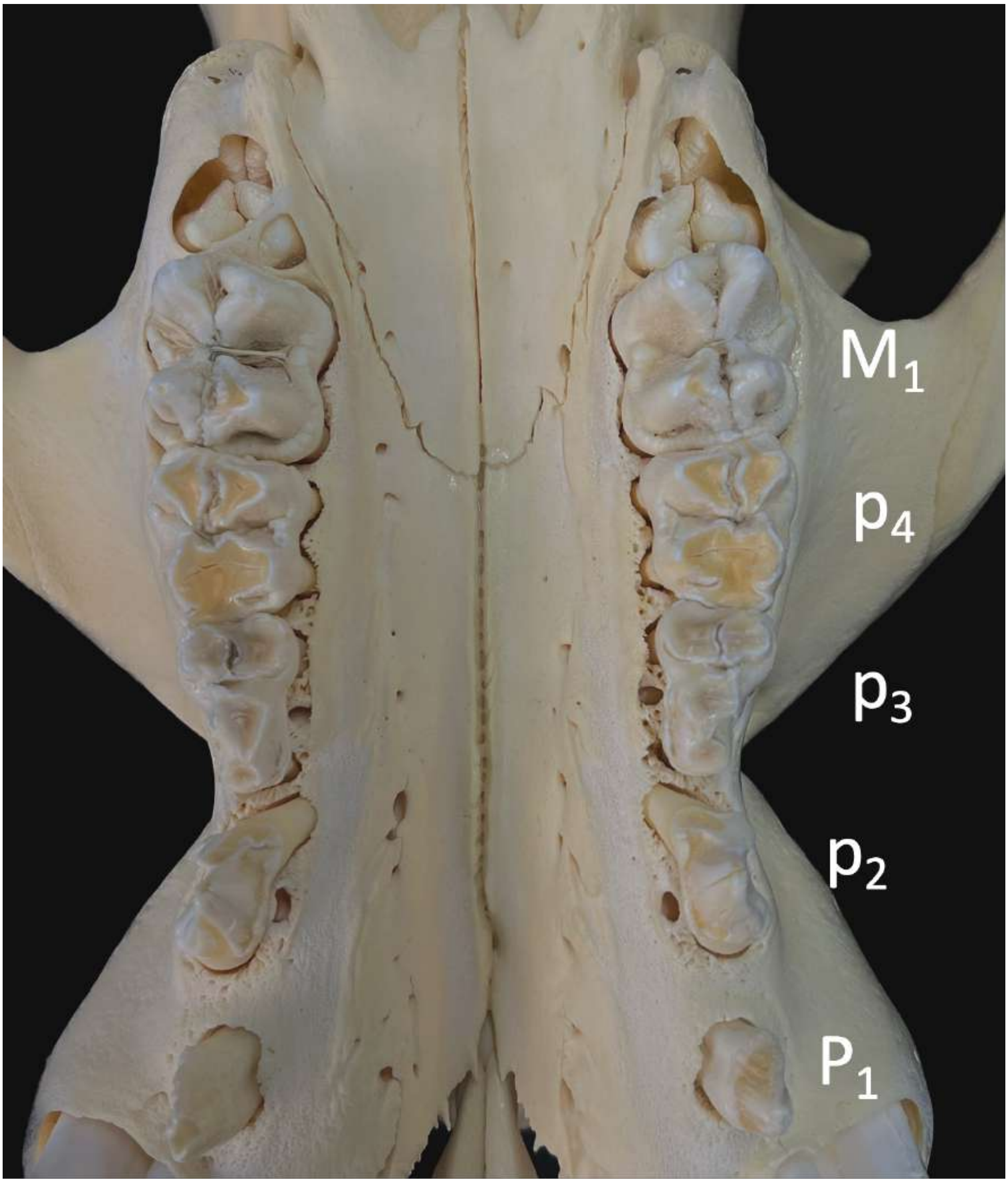

Figure S38 Maxillary milk dentition of a pygmy hippo (*Choeropsis liberiensis).* Photo: Annika Avedik.

Supplementary References

1. Shannon G, Sadler P, Smith J, Roylance-Casson E, Cordes LS. Contrasting selection pressure on body and weapon size in a polygynous megaherbivore. Biol Lett. 2021;17:20210368.
2. Matthes E. Abnorme Mandibularcanini bei Hippopotamus. Zschr Anat Entw Gesch. 1939;110:181-211.
3. Laws RM. Dentition and ageing of the hippopotamus. E Afr Wildl J. 1968;6:19-52.
4. Hooijer D. On the supposed hexaprotodont milk dentition in *Hippopotamus amphibius*. Zool Meded Mus Leiden. 1942;24:187-96.
5. Gomes Rodrigues H, Lihoreau F, Orliac M, Thewissen JGM, Boisserie JR. Unexpected evolutionary patterns of dental ontogenetic traits in cetartiodactyl mammals. Proc R Soc B. 2019;286:20182417.
6. Miles AEW, Grigson C. *Colyer's* Variations and diseases of the teeth of animals. Cambridge: Cambridge University Press; 2003.
7. Eltringham SK. The hippos: natural history and conservation. London: Academic Press; 1999.
