# Supplementary material for: Chewing, dentition and tooth wear in Hippopotamidae (Hippopotamus amphibius and Choeropsis liberiensis)
Source: PLoS One. 2023 Oct 4;18(10):e0291825. doi: 10.1371/journal.pone.0291825 (PMC10550173; doi:10.1371/journal.pone.0291825)
Supplement: Attachment — Submitted filename: Clauss_HippoTeeth_ReplyLetter_230814.pdf [file pone.0291825.s004.pdf]

Manuscript PONE-D-23-06724
Reply to Review

Dear Cyril Charles,

thank you for giving us the opportunity to revise our manuscript and respond to the referees' comments.

Please find attached the revised manuscript with all revised text in grey shading. Our response to specific referee comments are given at the bottom of this letter.

We made adjustments to the Materials and Methods section, and due to some comments of Reviewer 2, expanded the results and discussion (in both main text and supplement). We thank Reviewer 2 in particular for making us do this extra effort.
Please note that Reviewer 2 made on the other hand several comments that we do not wish to follow, and we give reasons for that in the tables below. This reviewer made several comments that appear as if neither the supplement, nor the appendix with the original data had been seen.

We thank you for your time and hope the manuscript now meets the requirements of PLoS One.

Sincerely,

Marcus Clauss

See detailed responses to reviewers down below

****

Referee 1:
This article is a thorough assessment of dental evidence relative to chewing movement in the two extant species of Hippopotamidae. This topic is of importance because it relates to dietary adaptations in a major group of megaherbivores characterized by a particular ecology, i.e. semiaquatic lifestyle, and with major bearing on African ecosystems – for the last five-seven years, many new papers explored the ecology of the common hippo, demonstrating its pervasive impact on continental water cycles and biodiversity. In addition, this study has implications for the evolutionary history of Hippopotamidae and of their anthracothere stem group, in which dietary changes (and notably grass consumption) co-occurred with increases of agonistic behavior. This paper provides further understanding of the anatomical trade-off between feeding and fighting that resulted in a successful expansion at the end the Miocene.
The paper is well-organized and well-written. Its illustrations are useful and clear. Above all, it includes a wealth of data. Notably, it provides for the first time a detailed assessment of anterior dentition occlusions and wear, and of their variations between individuals, hence going much further than the usual basic descriptions provided in literature. The study limits are well explained in the discussion, making the paper even more useful. Conclusions are sounds and congruent with results. This is overall a great contribution, and I believe it should be readily accepted. I just have spotted a few things that could be easily fixed as minor revisions.

Reviewer's comment (lines refer to the original submission)	Our response	Changes made in the text (lines refer to the revised version)
Line 92: ref [32] is not discussing phylogenetic relationships of pygmy hippos with other hippos. This would be rather in ref [42].	Our apologies, change made.	Citation changed (not marked) in l. 92.
Line 186: "Error! Reference source not found"<br>Please correct.	Our apologies for overlooking this error.	Change made l. 192: **Fig. S4** and **Fig. S5**
Fig. 13. For a more straightforward reading, each measurement position should be clearly indicated on this figure.	Ok, done	Figure changed (not marked)
Regarding cheek tooth measurements, from text and supplementary pictures it is not totally clear how these were taken: including cingula or not (when present), maximal width, or at wear figures? It would be useful to describe the measurement protocol with the same clarity as for anterior dentition. Also, were highly worn cheek teeth measured or not? Length of M1s is often significantly altered through wear, was this considered?	Fair point, we made the corresponding clarifications.	Addition made l. 196: All measures were performed on all teeth irrespective of wear status.<br><br>Addition made l. 198: maximal width (including the cingulum if present)
Line 405: "the general impression is that the dP4 and the M1 have wider posterior cusps, and the M3 a wider anterior cusp" This phrasing is a bit awkward: either this is true for all cases, or in most cases. For the latter it would be certainly easy to indicate the % of specimens this is not true.	Fair point. We refer to the corresponding Suppelementary Figures (S12-S16). We do not want to give the percentages, because this would mean that for each tooth position, we have to give the % for the mandibular and the maxillary one, for each species – this becomes a bit of confusing text for something that is of little value for the subsequent narration. If the editor wants, we can add this easily.	No change (unless editor rules)

Line 533. "We can only speculate that in incisors characterized by lateral or oblique facets, the tip will, under normal conditions, regularly break off or be worn down by using these teeth for digging and fighting, or manipulating hard objects". The authors should mentioned which hypothesis they favor here, based on literature and/or on their own observations. I have observed incisor breakages in several common hippos, and always assumed they were related to fight. Digging may indeed occur in Choeropsis liberiensis, but I am not sure we know how they proceed. Laws mentioned digging in Hippopotamus amphibius, but as far as I remember he is the only one and I did not read any account describing in details such digging. I do not recall any mentions of manipulating hard objects. If wear was caused by digging or hard object manipulating, it should have distinctive, recognizable patterns, and this could be tested.	We agree with the reviewer that our own (very limited! – only in zoos) observations were not on hard object manipulation, so we would also tend to "fighting". We added this, and also added the Laws citation (the only one we know, although this paper is cited for the "digging" function also by others.	Addition made l. 626: To our knowledge, no detailed observations on digging, or on object manipulation with incisors exist for hippos; therefore, fighting appears as the most likely cause. Observations in both free-ranging and zoo hippos on incisor use would be welcome.
Line 539 and following. The use of bicanine width compared in both species cannot be considered as a reliable inference of anisognathy, the latter being based on molars (e.g., Fortelius used M1s). Many hippopotamids differ in the relative expansion of the canine processes, and this projection can be much more readily seen as adaptive to agonistic behavior, little related to feeding, and thus largely uncorrelated with distance between molar rows. It would be more cautious to drop this point.	This is true, but please note we do not do that here. We cite literature, incl. Fortelius, about this measurement, we state at this very spot that we ourselves did not measure it (and we also stated that in the methods). We no longer call this measurement a 'proxy' now, and we give the definition of the Fortelius measurement.	Addition made l. 172: Unfortunately, the anisognathy index as described by Fortelius [1] was not measured; it is a ratio defined as the the buccal-to-buccal distance between a certain left and right molar position (typically, the M1) of the maxilla divided by the same distance of the mandible (both distances are corrected for the width of the corresponding tooth).
Lines 588-591. This is not totally clear. Dietary changes toward more grass eating have been actually recorded in conjunction with loss of schmelzmuster complexity. Co-occurrence of such dietary change with loss of lateral chewing movement may accompany lesser sensitivity to wear and greater susceptibility to tensile stress. These hypotheses are not mutually exclusive, but instead likely to reinforce each other.	Given that this is hypothetical, we would like to not use this argument by the reviewer.<br><br>1. We think that a change to grass AND a loss of lateral chewing movement is not something that occurred often, but rather quite rarely (because most large herbivores use some kind of transversal chewing movement).<br><br>2. We cite work that actually no rule of enamel schmelmuster complexity and diet seems to apply.<br><br>3. If, as the reviewer states, the move towards an orthal chewing movement should increase tensile stress (as opposed to our hypothesis that it reduces it, which we find more compelling because simple 'pressur' without a lateral compenonent does not sound like causing a lot of tensile stress), then how would this match the observation that the schmelzmuster of hippos appears particularly susceptible to tensile stress?	No change

****

Referee 2:
In this interesting study, the authors discussed the chewing capacity of the two extant species of hippos based on the direct observations of living animals, on measurements on dentition and on description of dental wear on died specimens (i.e. skulls).
The introduction and discussion are well written (even if there are a few digressions at the end of the paper p.29-30), and we understand that the main question is the chewing capacity of hippos, rather than a focus on dental anatomy, which is only complementary.

I have also minor comments and suggestions which are listed in the pdf file.

Reply: in the table below, we combined general statements with pdf file comments if applicable.

Reviewer's comment (lines refer to the original submission)	Our response	Changes made in the text (lines refer to the revised version)
The authors also studied young to adult specimens based on dental wear stages, but we have no information regarding age classes for each specimen (based on Laws' table or on relative macrowear stages defined by the authors); it should be detailed accordingly. This information on the relative age of specimens could be of high interest to compare the chewing capacity of young and adult specimens according to the relative development of anterior teeth and snout.<br><br>The relative age or ontogenetic stage can be determined using dental wear and dental eruption. It is seems important to differentiate young and adult specimens as the development of anterior teeth (especially canine) is different and could have a variable influence on the mastication (considering also the weaning age). It should have been tested. Maybe you already have some corresponding evidence or data.	But this is exactly what we did, as described in the methods – we estimated the age based on eruption and wear using the scheme of Law.<br><br>And for each individual, the age category of Law is given in the supplementary data file.<br><br>The question of when during ontogeny the lateral jaw movement is actually impeded is highly interesting! A very good comment, we tried to address it under 'limitations of the present study'.	Addition made l. 571: Finally, although some skulls of juvenile animals were included in our study (**Fig. S30-S38**), we did not assess at what age the constraining effect of the canines and the incisors should become pronounced. For example, the drawing of Laws [2] suggest that this might become particularly effective only at the age of 3-4 years in common hippos. Given that common hippos are weaned between one and two years of age [3], this would theoretically mean that there could be a short ontogenetic window in digestion of solid food supported by lateral chewing could occur. Whether hippos actually use this opportunity, or whether the general arrangement of the cranial skeleton and muscles nevertheless prevents this, remains to be investigated.
This study follows a recent publication about the same topic on mammals, in which the same authors only briefly introduced the case of hippos. In that respect, the authors should more clearly state what part of data shown in the present paper was already published.<br>I do not understand if the present results (and corresponding experiment) are new or from Avedik et al., 2023 (J. Morphol).	This comment refers to this statement in the text: "*We assessed the ranges of chewing motions with the museum skulls; the results of this evaluation have been presented in an abbreviated version in a review on how the anterior dentition can impede transversal chewing movements across mammalian herbivores* [4]".<br>We think this sentence clearly states that these results have been presented in the cited paper, albeit in an abbreviated version. We also mention that that paper is not about hippos specifically, but about basically all mammalian herbivores.<br><br>None of the measurements presented in this paper here was given in the previous paper – only qualitative observation which structures constrain the lateral chewing movement.	No change

Moreover, all measurements shown in figures must be presented in supplementary tables, and statistical tests are also needed for accurate comparisons (e.g. upper vs lower dentition).<br><br>Ok, but are these differences significant between upper and lower teeth. It should be tested.	We were surprised – we give all the original data in a supplement, which was also available to this reviewer (as evident on the last page of the pdf the reviewer annotated).<br><br>As to the statistics:<br><br>We had written that the anisodonty is evendent and referred to the data graph, which looks like this:<br>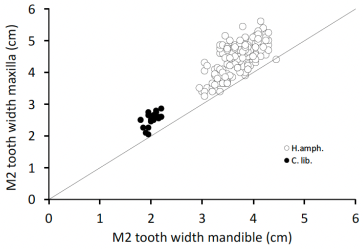<br>In other words, apart from a single specimen, the maxillary M2 is always wider than the mandibular one.<br>I was told repeatedly by statisticians that it is important to apply stats when it makes sense for the question. But these data are beyond doubt. We know how to run paired tests, but who would consider that necessary in this case?	No change (waiting for editor to rule)
Ok, but you did not measure it accordingly. I suggest to remove this statement.	But this is something from the literature, even if we did not measure it.	No change
anterior	ok	Addition made l. 29: in their anterior dental morphology
In Choeropsis	We are not sure what this comment implies. In this sentence, we say 'in theory', and this theory – which is about the canines and the snout – applies to both pygmy and common hippos. We explain in the subsequent sentences why, in the common hippo, additional limitations apply that prevent this theoretical possibility from being put into practice.	No change
Not necessary here	We ask the editor that we can leave this part of the sentence in. We have seen quite a lot of misplaced hippo incisors during our visits, and this is a finding of our study relevant for museum curators, so we want to leave it in the abstract.	No change
probably	Good point	Addition made l. 38: that was probably more prominent
Orthal movement exist in most mammals, because it is the condition of mammal ancestors. As a result, it can be only hypothesized that hippos loss the capacity of performing lateral movements, if they can only perform orthal movements.	This is a near philosophical point. We use the term "chewing", not the term "movement". We agree that all mammals have the capacity for orthal movement (nobody would doubt that), but it is the chewing pattern we refer to here – and that is something that not all mammals do – orthal chewing. Even if they have the capacity for orthal movement. So we think we should not change this here.	No change
Ok, but the dentition of old adults are frequently highly worn...	Yes, but that does not mean that the attrition is high, just the abrasion.	No change
pattern	Our apologies	Change made l. 44: pattern

Rather bunoselenodont	ok	Chang made l. 45: from an ancestral bunoselenodont condition
Can be used, but Cetancodonta is generally preferred.	Ok, we give both now	Addition made l. 72: classified under Cetancodonta or Whippomorpha
Only part of it (mainly anterior teeth).	This comment refers to our statement that we describe dental anatomy. We are not sure what the reviewer wants here? Nobody who investigates something will investigate 100% of all aspects. But to accommodate this statement, we added "aspects of".<br>But we do not do mainly the anterior teeth- we took measurements of the posterior ones as well.	Addition made l. 98: we aimed to describe aspects of chewing, dental anatomy, and tooth wear
Ok, but one can consider that data for upper teeth are finally scarce compared to Laws' data, if you did not take into account dental eruption and if you only showed wear stages for lower teeth (cf Fig. S7).	We do not understand this comment. We had written that we added "some" data for the upper jaw. Does the reviewer want us to say "only a few" here?<br>But to our knowledge, no measures for upper teeth exist for extant hippos, and we do give their wear stage (e.g. Fig. 17), not only the wear stage of the lower teeth, and also all the morphometrics.<br>We are at loss what to do with this comment.	No change
I am a bit confused by the "material and methods" section, which needs more details (e.g number of living animals filmed, what is direct observations, what is actually quantified).<br><br>You should precise how many animals were filmed and how many videos were recorded/used. Did you only use qualitative observations, or did you also quantify our observations? Add precisions please.	Good point. We added this to the videos.<br><br>For the museum skulls, we had even used a sub-heading "Qualitative observations".	Addition made l. 114: *H. amphibius*, we used a multitude of videos recorded at Copenhagen Zoo representing two individual hippos available via social media channels of Copenhagen hippo keeper Brian Stefanski.<br><br>Addition made l. 119: pygmy hippo, we made 22 videos of two animals at the Basel Zoo, the most useful being those when animals were handfed carrots<br><br>Addition made l. 124: These evaluations were or a qualitative nature, describing the movement of the jaw using various anatomical reference points; they do not represent quantitative data from a large number of individuals.
Did you take pictures for all specimens, for each phase of the chewing motion? It should be mentioned.	No we did not. We also did not do this in all specimens after the first 10 or so, but we did not note the number. This is why we use the subheading "qualitative observation" in the method section, and we added it again here.	Addition made l. 283: results of this qualitative evaluation
This is not the most conventional nomenclature because capital letters and lower-case letters usually refer to upper and lower dentitions respectively. You should instead use di, dc, and dp for deciduous teeth.	We do not think that there is a standard nomenclature that precludes our use of it – this is something one sees at least in the literature we are used to. For example, the Berkovitz book uses the nomenclature we use here.	No change
cranium	Good point	Change made l. 164: dimensions of the cranium

More frequently defined as the "anterior or rostral margin the premaxilla" and "posterior or caudal margin of palatine".	Good point, we added this.	Addition made l. 165: between the *Processus palatinus ossis incisivi* (rostral margin the premaxilla) and the caudal margin of the *Os palatinum* (caudal margin of palatine)
in	ok	Change made l. 170: measured: in the upper
What is the definition of this index and explain why you did not measure it accordingly ?	The definition is given now in the text; we did not measure it because we forgot to do so, very embarrassingly. But we thought we need to state this because otherwise readers might wonder why we do not report it.<br><br>We had already stated the 'unfortunately' in the method and in the discussion in the original submission to express that this was unfortunate – i.e. a mishap, not a plan.	Addition made l. 172: Unfortunately, the anisognathy index as described by Fortelius [1] was not measured; it is a ratio defined as the the buccal-to-buccal distance between a certain left and right molar position (typically, the M1) of the maxilla divided by the same distance of the mandible (both distances are corrected for the width of the corresponding tooth).

I am notably surprised that some measurements (e.g. cranial and mandible dimensions, gaps between premolars and between canines/incisors, height of cheek teeth) mentioned in the methods were not presented in the results (if we except supp. figures). If they are not relevant here (which seems to be the case), they should not be included in the paper.<br><br>You should more properly explain the interest of these measurements, these are unclear to me.<br><br>Did you actually use the dimensions of the cranium and mandible in your analyses?<br><br>What is the functional (i.e. occlusion) interest of considering the length of the tooth row without considering diastemata ? I do not think this is relevant.<br><br>You did not measure correctly the height of your cusp because you use the alveolar margin instead of the collar, to include only the crown and not the root (see Fig.S6AB). You should leave this measurement, not discussed below. Moreover, did you take into account wear stage for these measurements depending on the relative age?<br>What about dental height, if you actually measured it ?	The reviewer is correct here – these measures were not used in the first version of the manuscript. We had taken them to test how tooth row and diastema scale with skull size. We added these sections now to the methods, results, and discussion.<br><br>Cranium/mandible dimensions: Yes, they were already used, see e.g. Fig. S24. But we used it even more due to the following comments on our lack of use of the height data by this reviewer.<br><br>Diastemata: This was made to facilitate comparisons of actual tooth row length, as e.g. later done in this paper in Fig.15. We also used the diastemata information now.<br><br>We agree that we did not measure cusp height but tooth height, and reworded accordingly.<br><br>We use this measure now in the results section, relating it to age. In theory, tooth height should decrease with age (possibly compensated by cementum addition at the roots). This is discussed now.<br><br>We had intended to display this data at the beginning, but had omitted them from the original manuscript submission. Our apologies for this decision, which does not seem justified to us now – thanks to the reviewer's comments.	Change made l. 197: tooth height and maximal width (including the cingulum if present) at every cusp<br><br>Additions made l. 227: Several relationships were analyzed for their scaling pattern, using log-transformed values and linear regression in R [5], reporting the scaling exponent together with its 95% confidence interval (95%CI). If the 95%CI excludes 1.0, the respective scaling relationship is considered to be non-linear; the case of length-length relationships, deviation from linearity represents a deviation from geometric scaling [6]. Using the length of the cranium or mandible as the basis, we analyzed the scaling of the respective cheek tooth row (P2-M3) and the length of 'diastema' (the sum of the spaces between P3-P2, P2-P1 and P1-I2, or between P2-I2; using P1 if P2 not present). Similarly, we used the width of the cranium or mandible as basis and analyzed the scaling of the sum of all widths of the respective anterior dentition (I, C) and the sum of an distances between these teeth for their scaling.<br><br>Additions made l. 473: Plotting the upper and lower cheek tooth row length to the length of the skull and the mandible, respectively, in adult specimens, it is evident that variations in body size (as reflected in cranium or mandible length) do not translate linearly into variations in tooth row length within common hippos (i.e. there is negative allometry between tooth row length and skull length), whereas across the two species, this relationship is close-to-linear **(Fig. 17AB)**. Diastema length increases with the respective skull length at a more-than-linear scaling (positive allometry) within the common hippos, but for the mandible, linear scaling is not excluded across the two species **(Fig. 17CD)**. When comparing maxilla or mandible width to the width of anterior teeth or the space between the anterior teeth, most scaling relationships for the cranium included linearity within common hippos and across the two species, whereas for the mandible, the sum of anterior tooth width increased more-than-linearly with mandible width in common hippos whereas the space between the anterior teeth nearly stayed constant across body sizes; across both species, the tooth width scaling did not exclude linearity, but that of the interdental spaces was below linearity **(Fig. S27)**.<br><br>New figure addedl. 489: **Figure 17** Scaling relationships of the (A) maxillary and (B) mandibular cheek tooth row (P2-M3) or the 'diastema' space (any non-tooth space between the P3 and the closest incisor) for the (C) cranium and (D) the mandible in adult common hippo (*Hippopotamus amphibius*) and pygmy hippo (*Choeropsis liberiensis*). The scaling exponents (with 95%CI) of the grey line (across all individuals of both species) are (A) 0.87 (0.76,0.98), (B) 0.92 (0.84,0.99), (C) 1.50 (1.34,1.66), (D) 1.06 (0.89,1.23); of the dotted line (within *H. amphibius*) (A) 0.31 (0.09,0.53), (B) 0.48 (0.25,0.71), (C) 1.52 (1.07,1.98), (D) 1.62 (1.07,2.17). If the 95%CI of the scaling exponent is < 1, this indicates negative allometry, if it is > 1, it indicates positive allometry.<br><br>Addition made l. 500: Plotting tooth height against the age classes results in the expected pattern of a more or less distinct initial increase with age (representing tooth eruption) and a subsequent decrease of tooth height (representing wear) **(Fig. 18, Fig. S28-29)**. For both species, the individual specimens identified as zoo

		animals mostly had relatively high teeth for the estimated age classe (**Fig. 18, Fig. S28-29**). However, one must remember that age was not measured independently, but by the visual patterns of tooth eruption and wear.<br><br>Additional figure made l. 522: **Figure 18** Examples of the relationship of tooth height and estimated age class for the maxillary M1 in (A) common hippo (*Hippopotamus amphibius*); (B) pygmy hippo (*Choeropsis liberiensis*). For the full set of teeth, see **Fig. S28-29**.<br><br>Addition made l. xx: When using a macroscopic measure for wear, measuring the actual crown height (as opposed to measuring the height as done in the present study, as the distance to the bony alveolar margin) would have been a more insightful measure of crown tissue loss. Nevetheless, the shape of the height-age relationships of the present study **(Fig. 18, Fig. S28-29)** corresponds well to plots given in Fig. 13 of Laws [2] that relate tooth weight to age: during growth/eruption, there is an increase, followed by a decrease due to wear. To what degree the addition of cementum to the roots compensates for crown wear in hippos, as described in other mammals [7], is unclear. Laws [2] describes both, the addition of cementum layers and the resorption of dental roots with increasing age.<br>The observation that tooth row length **(Fig. 17)** does not scale as expected from geometry, but with negative allometry within the common hippos (in which a sufficient number of animals was available to test this), corresponds to similar observations within other species [6]. Actually, hippos have been part of the discussion on whether dwarf species retain relatively larger teeth compared to their ancestral, larger-sized relatives [8, 9]. Whether pygmy hippos actually have larger relative cheek teeth than common hippos depends on whether skull and mandible dimensions actually relate geometrically to body mass in these species [9] – a question that cannot be resolved with our dataset as body masses of the museum specimens had generally not been recorded. Nevertheless, the fact that the scaling between the two species tended to be of a negative allometry for the tooth row length but a positive allometry for the 'diastema' length corresponds to dwarfing effects among closely related species. As reviewed in [6], a mismatch in tooth size and body size is more likely the more recent the deviation of dwarfs or giants from the ancestral state in evolutionary time. The current interpretation of such findings is that tooth dimensions are more refractory to evolutionary changes in size than overall body size [6].<br><br>Addition made in Supplement: When comparing maxilla or mandible width to the width of anterior teeth or the space between the anterior teeth, most scaling relationships for the cranium included linearity within common hippos and across the two species, whereas for the mandible, the sum of anterior tooth width increased more-than-linearly with mandible width in common hippos whereas the space between the anterior teeth nearly stayed constant across body sizes; across both species, the tooth width scaling did not exclude linearity, but that of the interdental spaces was below linearity **(Fig. S27)**. This indicates that in common hippos, the lower first incisors and/or canines increase with positive allometry with mandible width.

		Plotting tooth height against the age classes reflects tooth wear **(Fig. S28-29)**.<br><br>Additional Figures made in Supplement:<br>**Figure S27** Scaling relationships of the width of the (A) maxillary and (B) mandibular anterior teeth row (I,C) or the interdental space between them for the (C) cranium and (D) the mandible in adult common hippo (*Hippopotamus amphibius*) and pygmy hippo (*Choeropsis liberiensis*). The scaling exponents (with 95%CI) of the grey line (across all individuals of both species) are (A) 0.91 (0.75,1.08), (B) 1.04 (0.93,1.15), (C) 1.19 (1.09,1.30), (D) 0.76 (0.61,0.90); of the dotted line (within *H. amphibius*) (A) 1.17 (0.79,1.35), (B) 1.49 (1.16,1.82), (C) 1.07 (0.79,1.35), (D) 0.18 (-0.25,0.60). If the 95%CI of the scaling exponent is < 1, this indicates negative allometry, if it is > 1, it indicates positive allometry.<br>**Figure S28** Tooth height in relation to the estimated age class in common hippo (*Hippopotamus amphibius*) of known (wild, zoo) or unknown origin.<br>**Figure S29** Tooth height in relation to the estimated age class in pygmy hippo (*Choeropsis liberiensis*) of known (wild, zoo) or unknown origin.

It should be modified.	Our apologies for overlooking this error.	Change made l. 192: **Fig. S4** and **Fig. S5**
You rather estimated the area, because this is not the outlined area.	Good point	Change made l. 201: tooth area was estimated
Ok, it seems that you mainly focused on dental wear stages than on dental eruption, as detailed by Laws, while it is of interest. But you should have considered that the dental eruption patterns are slightly different between these species (see Gomes Rodrigues et al., 2019, Unexpected evolutionary patterns of dental ontogenetic traits in cetartiodactyl mammals Proc. R. Soc. B.). Why did you not use such age categories to discriminate young and adult specimens?	We do not understand the comment.<br><br>1. The scheme by Laws, which we present in the Supplementary material, uses both, dental eruption and dental wear stages. Evidently, using only eruption would not cover the later age classes.<br><br>2. No similar scheme is available for pygmy hippos.<br><br>3. We had been aware of Gomes Rodrigues et al. (2019), but we did not see from the main paper that that paper provided more detail than Laws in terms of eruption sequence. Fig. 2 of that paper indicates the same eruption sequence (P4 before M3) for both species. Given the comment of the reviewer, we checked the paper again and found,<br>(i) this sentence: "*M2 erupts before P2 in most studied cetartiodactyls, while in extant hippopotamids, it erupts at the same time (Choeropsis) or after P2 (Hippopotamus).*"<br>(ii) in the supplement, a more detailed eruption sequence for both species that actually shows even some more subtle differences.<br><br>We added this to the text in the supplementary materials now incl. the citation. However, this data does not give any indication of age classes – it is just the bare eruption sequence without any link to age, and no relative eruption sequence across upper and lower jaw.	Addition made in Supplement l. 38: More recently, the full eruption sequence for common and pygmy hippos have been published in the supplementary material of Gomes Rodrigues et al. [10].<br><br>*Hippopotamus amphibius*<br>Upper jaw: $dc\text{-}dp^2\text{-}dp^3$ $(\rightarrow)$ $dp^1 \rightarrow dp^4 \rightarrow C,M^1 \rightarrow P^2 \rightarrow P^3,M^2 \rightarrow M^3$ $(\rightarrow)$ P^4 [10]<br>Lower jaw: $dc \rightarrow dp_1,dp_2,dp_3$ $(\rightarrow)$ $dp_4 \rightarrow C \rightarrow M_1 \rightarrow P_2 \rightarrow P_3,M_2 \rightarrow P^4$ $(\rightarrow)$ M^3 [10]<br>$dc,dp_1,dp_2,dp_3 \rightarrow dp_4 \rightarrow C,M_1 \rightarrow P_2 \rightarrow P_3,M_2 \rightarrow P^4$ $(\rightarrow)$ M^3 [11]<br><br>*Choreopsis liberiensis*<br>Upper jaw: $dc \rightarrow dp^2\text{-}dp^3 \rightarrow dp^1,dp^4 \rightarrow C,M^1 \rightarrow M^2 \rightarrow P^1,P^2,P^3 \rightarrow P^4 \rightarrow M^3$ [10]<br>Lower jaw: $dc,dp_2,dp_3,dp_4 \rightarrow C,dp_1,M_1 \rightarrow P_2,M_2 \rightarrow P_3 \rightarrow P^4$ $(\rightarrow)$ M^3 [10]
If you did not consider it at all, you should leave this part (including supp material).	But we do report it in the Supplementary material and also explain it there. Why should that not be possible or good? Does the reviewer suggest we rather delete this than have it in the Supp.?	No change
You should explain more precisely how these evaluation are conducted.	We thought this was evident, but we put it into distinct words now.	Addition made l. 247: The mastication of the common hippo was evaluated on the feeding videos, by observing whether a lateral component of mandibular movement could be detected, using the various anatomical structures of the upper and lower jaw as reference points
Delete "mesial" from Fig. 1 legend	agreed	Deletion made l. 269: revealing a non-natural cutting surface
Are differences between facets less marked in young specimens of Choeropsis?	Good point. The reviewer is correct. This was added to the figure legend.	Additioin made l. 300: Note the general similarity of facet widths in common and the generally larger maxillary facet width in older pygmy hippos
If it is the case in both young and adult specimens, it must be clearly stated.	This is a good point. Fig. 5 actually answers that question, but the reviewer is right, we can write it out clearly.	Addition made l. 313: In both species, the lower canine was longer than the upper one, irrespective of the age of the specimen (**Fig. 5**)

I do not really understand this sentence. It should be removed.	Rather than removing it, we would rather explain it better.	Change made l. 424: the general impression is that the dp4 and the M1 have wider posterior cusps, and the M3 a wider anterior cusp; this most likely reflects the width of their respective osseous bases of the maxilla or mandible at the time of tooth formation
If the dark lines represent standard deviations, it should be noted.	Good point	Addition made l. 434: Data only from specimens with all teeth. Dark lines represent the standard deviation.
I do not think this is of high interest if it is not further discussed.	It is not further discussed, but it is an appropriate and correct description of the anatomy.	No change
Same thing for dark lines.	Good point	Addition made l. 457: Data only from specimens with all teeth. Dark lines represent the standard deviation.
This means that wear is intense, despite the limited lateral chewing movement...	Yes, it does. Yet, we do not know what this comment implies, because we never claim there is no wear – actually, right at the spot of the comment, we explain the wear as a result.	No change
Do you know any evidence or references on extinct hippopotamids supporting your hypothesis ?	This is the first paragraph of the discussion. We give all the evidence and all the references we know in the subsequent sections.	No change
These configurations should have been depicted in supplementary data.	This means the reviewer asks that all these figures from the literature: Fig. 18-19 of [47], Fig. 8-11 of [48], Fig. 629 of [5], Fig. 3-4 of [44], Fig. 12c of [49], Fig. 2 of [50], Fig. 2 of [15] should be reproduced in the supplement? We will do this if the editor rules we should.	No change (but will do if editor rules)
Ok, but you have different wear stages/ontogenetic stages, and you could use it for your interpretations.	We do not understand this comment – this would not help in identifying zoo from free-ranging animals	No change
Their tip are however highly worn for most of them (see fig. 9-12, not only lateral facets)...	But this is not the issue in this sentence, it is whether that wear is caused by an antagonist tooth or not. The following sentence makes this even clearer.	No change
You should add some precisions or cite some examples of moderate or strong anisognathy in ungulates for a more accurate comparison.	Good point. We added other ungulate data.	Addition made l. 642: These values are comparatively low among ungulates; for example, clearly laterally chewing ruminants, equids or rhinoceroses have anisodonty ratios of 1.5-2.0 [1]. This supports the
The unique orthal movement can be a secondary evolutionary feature.	We do not understand what the reviewer wants here. Our text says exactly this: we hypothesize that the orthal chewing mode is a secondary feature.	No change
Explain why?	That is exactly what is done in the next sentences …	No change
Radial enamel is also more efficient to withstand high wear.	This may well be the case (although the reviewer does not give a citation), but we are concerned with chewing directions here, not with whether wear is “high” or “low”.	No change
bunoselenodont	Good point	Change made l. 699: from bunoselenodont ancestors

But is it also true for highly derived anthracotheriids, such Libycosaurus and Merycopotamus, whose cranio-dental specializations are close to hippopotamids? These taxa are worth to investigate in that respect.	Very good point. We added this question (as we are not aware of an answer).	Addition made l.717: It would probably be worthwhile determining these indices for a variety of hippopotamid ancestors and fossil relatives for which cranium and mandible are conserved for a specimen, and put these findings into context with the occlusal surface pattern.
It is much more like that. In fact the whole body of hippopotamids is highly specialized for semi-aquatic life, and this evolutionary trend is already encountered in anthracotheriids.	It is not clear how the reviewer wants us to change the text here. In the context of the narrative, we believe our sentence is valid.	No change
Not italicised	Good point	Changed (not marked)
